# Recent Advancements in Ambient‐Air Fabrication of Perovskite Solar Cells

**DOI:** 10.1002/EXP.20240121

**Published:** 2025-03-06

**Authors:** Yihuai Huang, Wenguang Zhang, Yuchen Xiong, Zijun Yi, Changkai Huang, Qinghui Jiang, Abdul Basit, Guibin Shen, Yubo Luo, Xin Li, Junyou Yang

**Affiliations:** ^1^ State Key Laboratory of Material Processing and Die & Mould Technology Huazhong University of Science and Technology Wuhan P. R. China; ^2^ China‐Eu Institute for Clean and Renewable Energy Huazhong University of Science and Technology Wuhan P. R. China; ^3^ Department of Applied Physics The Hong Kong Polytechnic University Kowloon Hong Kong; ^4^ School of Electrical and Electronic Engineering Nanyang Technological University Singapore Singapore

**Keywords:** ambient air fabrication, degradation mechanism, enhanced stability, large‐scale, perovskite solar cells

## Abstract

Perovskite solar cells (PSCs) have attracted considerable attention due to their potential for high‐efficiency conversion and cost‐effective fabrication. Although the fabrication of perovskite films in ambient air offers environmental and cost advantages, the presence of water vapor and oxygen may induce instability in these films, thereby affecting device performance. This review aims to comprehensively explore recent advancements in the fabrication of PSCs in ambient air, while investigating various factors contributing to perovskite degradation. Addressing these challenges, diverse fabrication strategies are outlined, encompassing compositional, additive, solvent, and interface engineering to enhance the performance and stability of PSCs fabricated under ambient air. To facilitate the commercialization of PSCs, this paper summarizes several widely employed methods for the large‐scale manufacturing of PSCs. Through this review, we aim to offer some invaluable insights and guidance for the commercialization trajectory of PSCs, as well as the pros and cons to their widespread applications in the field of renewable energy.

## Graphical abstract

This review explores the fabrication of perovskite solar cells (PSCs) in ambient air, highlighting strategies to enhance their stability and performance. It discusses challenges such as degradation under environmental conditions and outlines methods for large‐scale manufacturing, offering insights into the commercialization and future development of PSC technology for sustainable energy solutions.

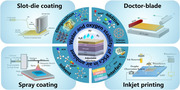

## 1 Introduction

The surge in energy crisis and critical challenges by climate change have emerged a great interest in the development of renewable energy in the past decades [1]. Being a sustainable and inexhaustible energy resource, solar energy not only ensures abundant, cost‐effective, and eco‐friendly power, while impending to meet the surging global energy demands [2]. In this regard, photovoltaic (PV) systems governed by the photovoltaic effect in semiconductors facilitated the direct conversion of solar radiation into electrical energy. For example, first‐generation photovoltaic technology based on crystalline silicon has engaged the share market up to 90% of photovoltaics globally [3]. In addition, silicon‐based solar cells have demonstrated an impressive efficiency ≈26.81% that is close theoretically to the Shockley–Queisser (S‐Q) limit ≈29.4% [4]. Though high costs and complicated manufacturing have greatly impeded their industrialization and commercial adoption, therefore, the third‐generation new thin‐film solar devices, including metal halide perovskite solar cells (mh‐PSCs) have gained significant attention in contrast. So far, mh‐PSCs have resulted in exceptional photoelectric properties, which were attributed to their large absorption coefficient, low exciton binding energy, high carrier mobility, etc. [5] Perovskite materials feature an ABX_3_ chemical structure with monovalent cation A‐site that is, CH_3_NH_3_ (MA)^+^, CH(NH_2_)_2_ (FA)^+^, and CS^+^, however, the divalent metal cation (Pb^2+^, Sn^2+^) and halogen anion ($Cl^{-}$, $Br^{-}$, and $I^{-}$) were considered at B and X‐site, respectively [6]. To date, several efforts have revealed a significant enhancement from 3.8% to 26.1% in the power conversion efficiency (PCE) of single‐junction PSCs since 2009, indicating a record performance of monocrystalline silicon solar cells [7]. From the above, one can easily elucidate that the advancement of perovskite materials in solar energy has opened up new opportunities for sustainable energy solutions, thereby a detailed study in the below sections suggests the PSCs as potential candidates for green energy.

**FIGURE 1:**
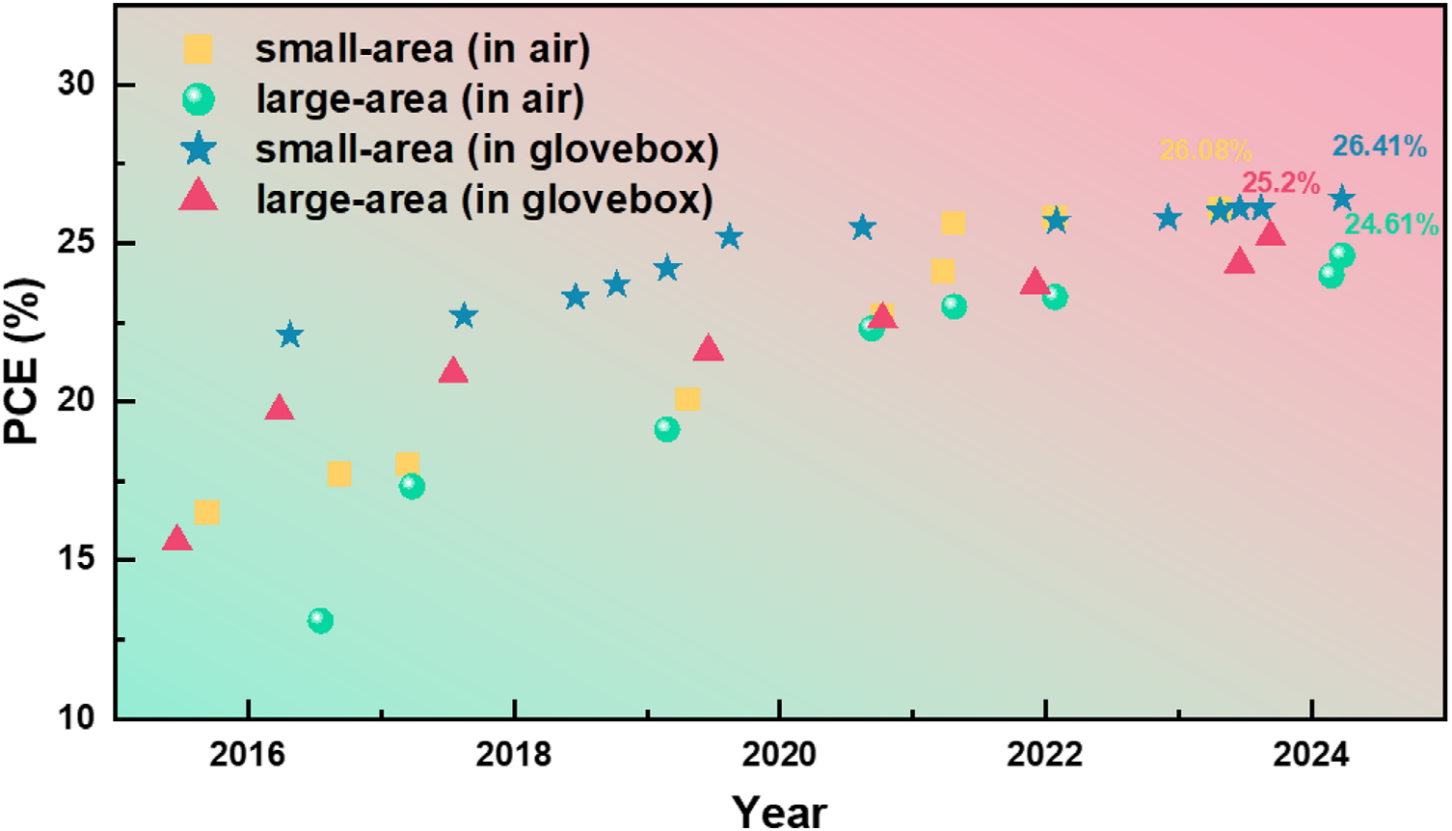
Chronological evolution of champion PCE for small‐area and large‐area PSCs in air and glovebox. (Small‐area and large‐area refer to active area about 0.1 and 1 cm^2^, respectively).

Despite the consistent efforts to attain high efficiency in PSCs, progress in enhancing the stability of these devices was relatively slow. For example, the preparation of efficient PSCs in a humid environment led to the major challenge attributed to the inherent instability under ambient conditions [8]. Reportedly, the majority of high‐efficient PSCs are derived from lab‐scale small‐area devices, typically with dimensions <0.1 cm^2^, fabricated by spin‐coating in an inert atmosphere under nitrogen or pure argon atmosphere [6, 9]. Although spin‐coating within a glovebox is operationally straightforward and offers satisfactory reproducibility, it lacks scalability for large‐scale production of PSCs [10]. In contrast, ambient air processing has acknowledged a critical technology that led laboratory‐scale to industrial‐scale manufacturing, and thus, several moderate strategies and fabrication methods contributed to the high‐quality development of PSCs recently [11]. For example, slot‐die coating, screen‐printing, spray‐coating, inkjet‐printing, and so forth, have been applied to large‐scale photovoltaic devices [12]. Despite these advancements, reports indicate that achieving significant industrial performance with large‐area devices remains a challenging and demanding task [13]. In pursuit of highly stable perovskites, various strategies have been employed, including compositional, additive, solvent, and surface engineering. These efforts aim to enable the industrial‐scale production of PSC technology in the renewable energy sector. In this paper, we have summarized the major challenges in the development of PSCs in ambient air. Over recent years, the performance of both small‐area and large‐area PSC devices fabricated in ambient air and glovebox environments has been depicted in Figure 1. This paper may help to understand the underlined perspectives to attain the highly stable PSCs for large‐scale commercialization. The main debates includes: (1) factors affecting the preparation of perovskite films in ambient environments, (2) favorable strategies to ensure the stability of PSCs, (3) methods for large‐scale preparation, and (4) prospects for the future development of PSCs.

## 2 Degradation Issues of Perovskites in Ambient Air

High‐quality perovskite films, characterized by high surface coverage, large grain sizes, low defect concentrations, and consistent crystal orientation, constitute a fundamental requirement for the stability and efficiency of PSCs [14]. Typically, these films are synthesized within a meticulously controlled glovebox environment under inert conditions. Despite notable advancements where researchers have successfully fabricated efficient and durable PSCs in ambient air, achieving versatility for various applications remains a persistent challenge. Several common factors, including moisture and oxygen, originating from the perovskite precursor, spin coating process, and annealing steps, significantly impact the crystallization growth, orientation, and structural integrity of perovskite films [8, 15]. Therefore, a comprehensive understanding of the influence of water and oxygen on perovskites during the preparation of high‐efficiency large‐area PSCs in ambient air is elaborated in the following section.

### 2.1 Water‐Induced Perovskite Degradation

The most familiar perovskite absorber CH_3_NH_3_PbI_3_ (MAPbI_3_) used in solar cells is typically synthesized through the mixture of methylammonium iodide (MAI) and lead iodide (PbI_2_) under an inert atmosphere condition (Equation (1)), however, the degradation process of perovskite films is accelerated by the high hygroscopicity of the methylammonium (MA) cation [16].

(1) $$CH_{3}NH_{3}I+\mathrm{Pb}I_{2}↔CH_{3}NH_{3}\mathrm{Pb}I_{3}$$

**FIGURE 2:**
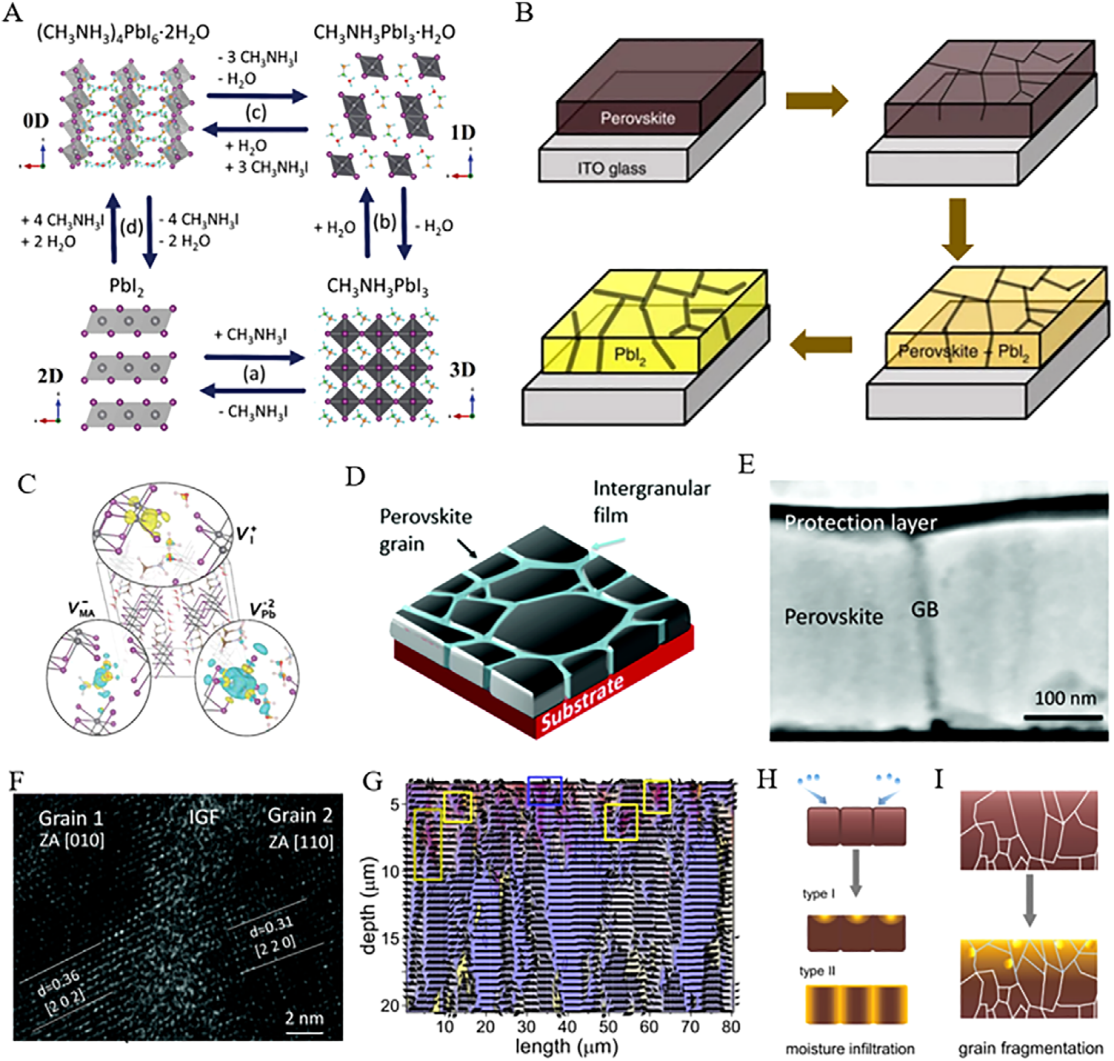
(A) Schematic illustrating phase equilibria in the PbI_2_‐CH_3_NH_3_I‐H_2_O system. Reproduced with permission [19]. Copyright 2016, John Wiley and Sons. (B) Schematic illustrating perovskite material degradation processes. Reproduced with permission [22c]. Copyright 2016, Nature. (C) Isosurface plot showing electronic charge density changes due to the formation of vacancy point defects $V_{I}^{+}$, $V_{\mathrm{MA}}^{-}$, and $V_{\mathrm{Pb}}^{-2}$ in MAPbI_3_·H_2_O. Reproduced with permission [26]. Copyright 2018, American Chemical Society. (D) Schematic showing intergranular film at grain boundaries. Reproduced with permission [28c]. Copyright 2017, RSC. (E) A cross‐sectional STEM (scanning transmission electron microscopy) image of the perovskite sample. Reproduced with permission [28c]. Copyright 2017, RSC. F) High‐resolution transmission electron microscopy (HRTEM) cross‐section image revealing the intergranular film between adjacent grains. Reproduced with permission [28c]. Copyright 2017, RSC. (G) Overlap of (100) perovskite peak orientation map with PbI_2_ (100) peak intensity map. Blue and yellow boxes highlight perovskite degradation at the grain surface and boundary, respectively. Reproduced with permission [29]. Copyright 2022, Springer Nature. Schematic illustration depicting (H) the moisture‐induced degradation of the perovskite film and (I) the fragmentation of grains within the degradation region. Reproduced with permission [29]. Copyright 2022, Springer Nature.

The above equation understands that moisture may easily infiltrate CH_3_NH_3_PbI_3_, resulting the structural instability at low humidity [17]. In this regard, first‐principles calculations demonstrated the adsorption energy of water molecules ≈0.30 eV on the (001) plane of CH_3_NH_3_PbI_3_, however, the considerable interspace corresponding to CH_3_NH_3_PbI_3_ may cause the penetration by water molecules that resulting structure corrosion [18]. Further, Song et al., precisely uncovered the degradation mechanism of CH_3_NH_3_PbI_3_ at a microscopic scale by laser beam, aiding in enhanced long‐term stability [19]. Based on the different exposure conditions, the degradation of CH_3_NH_3_PbI_3_ can be categorized into four stages, including three reversible stages and one irreversible degradation stage, mainly by evaporation of organic components [19]. For instance, Figure 2A illustrates the synthesis (forward) and thermal decomposition (reverse) reactions of CH_3_NH_3_PbI_3_ in a moisture‐free environment according to Equation (1) [19]. Further, CH_3_NH_3_PbI_3_ undergoes a hydration reaction (according to Equation (2)) after the introduced trace water, resulting in the production of 1D monohydrate ($CH_{3}NH_{3}\mathrm{Pb}I_{3}\cdot H_{2}O$). Leguy et al. further demonstrated the formation of an aqueous phase that, independent of the film's depth, indicates rapid water transport along grain boundaries.[20] Similarly, Equation (3) suggests the formation of a smaller 0D dihydrate phase (${\left(CH_{3}NH_{3}\right)}_{4}\mathrm{Pb}I_{6}\cdot 2H_{2}O$) in a 1D monohydrate chain reaction under a concentrated water atmosphere. This phenomenon requires a significant amount of CH_3_NH_3_ cations that yield a by‐product PbI_2_ [19], while organic and inorganic components redistribute and subsequently lead to the variation of grain volume and film morphology. On the other side, Equation (4) is the irreversible conversion of dihydrate to volatile CH_3_NH_3_I as well as the residue PbI_2_, suggesting the complete degradation of CH_3_NH_3_PbI_3_ [19]. Such degradation was further confirmed by Yang et al. [21], through water‐induced CH_3_NH_3_PbI_3_ under light exposure, as characterized by a color transition from deep brown to yellow (see Figure 2B). This approach not only leads to a sudden deteriorated performance while implying some alarming concerns from the low solubility of PbI_2_ in water as well as some toxic risks [22].

(2) $$CH_{3}NH_{3}\mathrm{Pb}I_{3}+H_{2}O↔CH_{3}NH_{3}\mathrm{Pb}I_{3}\cdot H_{2}O$$

(3) $$\begin{array}{c} \left(4-n\right)CH_{3}NH_{3}\mathrm{Pb}I_{3}+\mathrm{nC}H_{3}NH_{3}\mathrm{Pb}I_{3}\cdot H_{2}O+\left(2-n\right)H_{2}O\\ \;↔{\left(CH_{3}NH_{3}\right)}_{4}\mathrm{Pb}I_{6}\cdot 2H_{2}O+3\mathrm{Pb}I_{2} \end{array}$$

(4) $${\left(CH_{3}NH_{3}\right)}_{4}\mathrm{Pb}I_{6}\cdot 2H_{2}O\to \mathrm{Pb}I_{2}+4CH_{3}NH_{3}I+2H_{2}O$$

(5) $$\mathrm{CsPb}I_{3}\to Cs^{+}+I^{-}+\mathrm{Pb}I_{2}$$

Recent reports demonstrate the intricate interplay between the deterioration of moisture‐induced perovskite and the emergence of surface crystal defects (i.e., vacancies and lattice distortions) [23], however, the surpassed adsorption energy of water molecules alarming the damage of perovskite structure at vacancy sites [24]. In this regard, computational simulations by Mosconi et al., [25] have well‐elucidated the interaction between the surface of MAPbI_3_ and water molecules, revealing the interaction between water molecules and released I atoms by Pb sites culminated in the desorption of MA molecules and the solvation of MAI surface. In contrast, the formation of Pb─I bonds leads to greater stability in humid environments of PbI_2_ terminal surface. Turning to the introduction of PbI_2_ vacancies, PbI_2_ terminal surface triggered the formation of water‐soluble Pb species and resulted in a swift dissolution of the entire perovskite material [25]. In the same fashion, Kye et al., reported [26] a substantial reduction in kinetic barrier for I migration during the hydration process that adopted the creation of PbI_2_ vacancies. Besides PbI_2_ vacancies, Figure 2C shows some point defects where I and MA were readily engendered in the aqueous phase [26]. Thus, deep energy levels corresponding to the charge transfer induce and hence cause the degradation of perovskites. Surface halide vacancies in perovskites facilitated the migration of $I^{-}$ and $Br^{-}$ ions as assisted by water and effectively form Br‐rich and I‐rich phases due to their distinct affinities (Pb^2+^, $I^{-}$ and $Br^{-}$) [27]. Consequently, water‐induced phase separation occurred through a common process of halide vacancies and ion migration, while perovskite grain boundaries also facilitated a rapid infiltration of water and participated in the degradation process [28]. Similarly, Ahn et al., [22c] observed the charge capturing at grain boundaries that trigger the irreversible degradation of moisture‐induced perovskites, for example, the grain boundaries in CH_3_NH_3_PbI_3_ featuring amorphous non‐crystalline films (see Figure 2D–F) due to the absorption or reactivity of water molecules [28c]. Moreover, the enlarged degradation along the in‐plane direction of grain was attributed to the water penetration into the crystal interior, and hence resulted in large clusters as shown in Figure 2F,G [29]. For instance, in Figure 2H,I illustrated that water‐induced degradation leads to grain fragmentation due to stretching strain in the upper region [30]. This phenomenon suggests that the rate of degradation is correlated with grain size, for example, a smaller grain experiencing more impact [28, 31].

**FIGURE 3:**
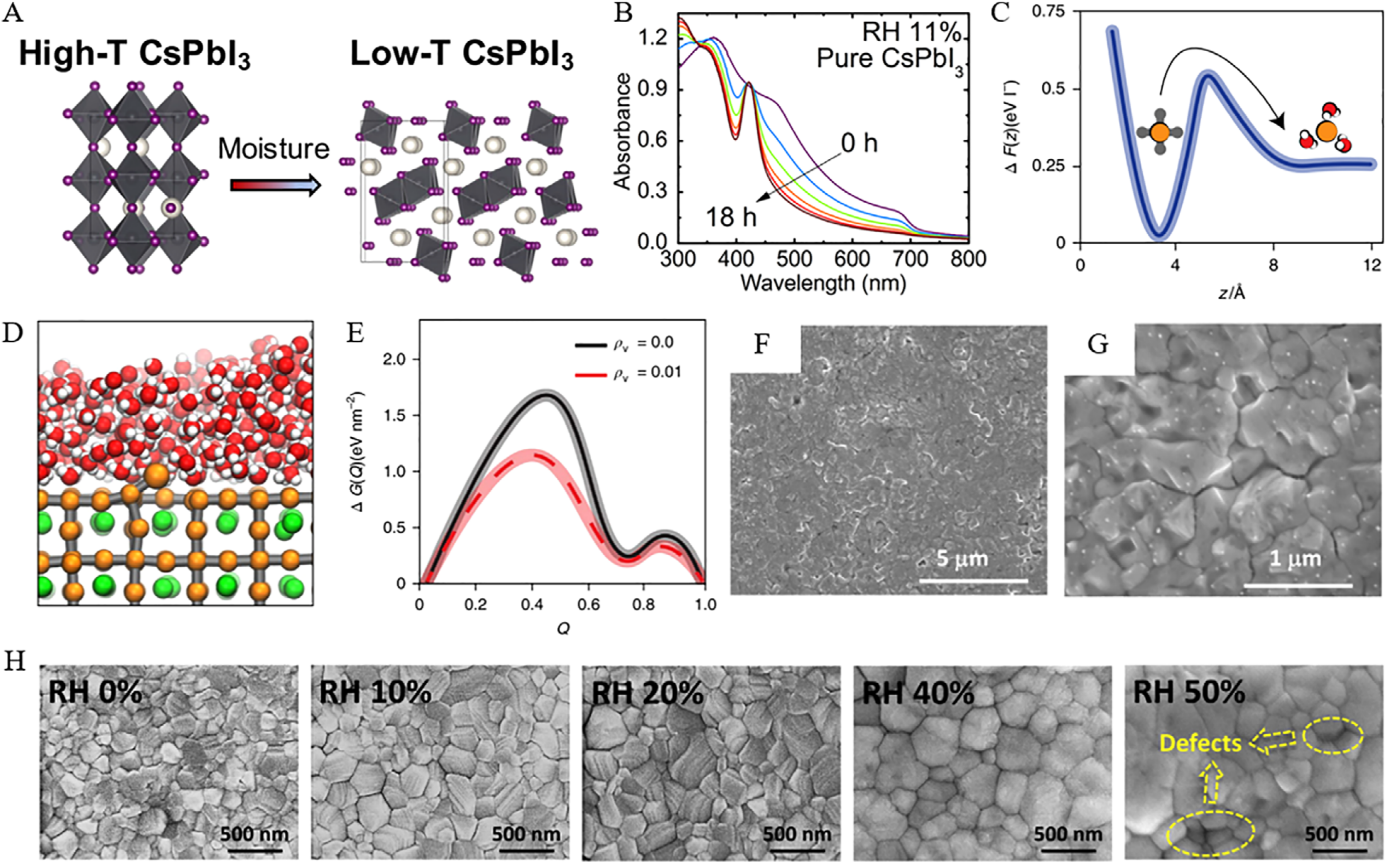
(A) Schematic showing the high‐T CsPbI_3_ phase (left, 3D corner‐sharing octahedra) to low‐T CsPbI_3_ (right, 1D chains of edge‐sharing octahedra) phase transformation triggered by moisture. Reproduced with permission [33]. Copyright 2021, Elsevier. (B) Spectra data collected over 18 h for pure CsPbI_3_ samples exposed to 11% RH at 23°C. Reproduced with permission [34]. Copyright 2016, American Chemical Society. (C) Reversible work, ∆*F(z)*, to transfer an $I^{-}$ atom from perovskite to the adsorbed water layer. Reproduced with permission [32b]. Copyright 2018, Springer Nature. (D) Molecular dynamics simulation snapshot near the barrier top in (C). Reproduced with permission [32b]. Copyright 2018, Springer Nature. (E) Free energy diagram depicting the transformation from high‐T to low‐T phase (∆*G(Q)*). Reproduced with permission [32b]. Copyright 2018, Springer Nature. (F,G) Scanning electron microscope (SEM) images of perovskite film post‐annealing in ambient air. Reproduced with permission [14b]. Copyright 2014, AIP. (H) Top‐view SEM images of perovskite films show a comparison of grain size with processing humidity ranging from 0% to 50%. Reproduced with permission [41]. Copyright 2021, Elsevier.

Majorly, inorganic halide perovskites tackled some drastic challenges of moisture instability, particularly the phase transition induced by humidity. For example, the transformation of CsPbI_3_ films from *α* to *δ* phase induced by moisture has been identified as a major cause of deactivation [32]. In this scenario, Figure 3A reveals that moisture erosion has triggered the transition of CsPbI_3_ from the metastable high‐temperature perovskite phase to the low‐temperature non‐perovskite phase, thus leading to a dramatic reduction in the conversion efficiency of CsPbI_3_ [33]. In addition to the moisture adsorbed on the surface of perovskite films, Dastidar et al., [34] found vacancies in the lattice, and hence nucleation‐free energy barriers were reduced and led to the new phase comprising a strong absorption peak (see Figure 3B). In contrast, the reduced free energy barrier of halide vacancies resulted in an exponential increase of the transition rate [32b]. Further, a significant increase in halide vacancies upon the contact of the adsorption layer with an interface was investigated through free energy calculations of CsPbI_3_ as illustrated in Figure 3C,D [32, 35]. Herein, the computed results of free energy (Figure 3E) suggest the increase in $I^{-}$ vacancies induced by moisture, which can be attributed to the large solvation enthalpy of halide ions and their low vacancy formation energy [32, 36]. Another degradation mechanism for CsPbI_3_ through thermodynamic calculations was reported, where the hydrolysis of CsPbI_3_ ultimately forms Cs^+^, $I^{-}$, and PbI_2_ according to Equation (5) [37]. The computational simulations of water adsorption on the surfaces of CsPbI_3_ and MAPbI_3_ have further confirmed the superior moisture stability of CsPbI_3_ [38]. The moisture‐induced phase transition in inorganic perovskites deviates significantly from the degradation process as observed in mixed perovskites.

Referring to the sensitivity of perovskites to moisture, trace amounts of water have been found to effectively promote recrystallization at grain boundaries and passivation of trap states. This effect may enhance the system morphology and yield a high‐quality film [39]. Therefore, Yang et al., [14b] proposed high crystallinity of perovskite films in a humid environment through a wet air annealing process, and thus an increase in grain size and surface defects were realized (see Figure 3F,G) [14b]. On the other hand, the controlled moisture treatment of intermediate perovskites by Liu et al., facilitated a substantial migration of organic salts into buried regions [40]. This phenomenon revealed the reaction between organic salts and PbI_2_ resulted in a uniform perovskite phase with larger grains and higher crystallinity. Similarly, another effort by Huang et al., [41] precisely investigated the material transformation and crystallization of perovskite films during annealing using in situ synchrotron X‐ray characterization. They found enhanced grain size and transformation rate by the inclusion of water, thus, the perovskite crystal orientation, lattice constant, and grain size were identified during annealing. Particularly, perovskite crystals have proven a preferred orientation, rapid annealing kinetics, and maximum grain size followed by the annealing with 40% humidity (Figure 3H).

### 2.2 Oxygen‐Induced Perovskite Degradation

**FIGURE 4:**
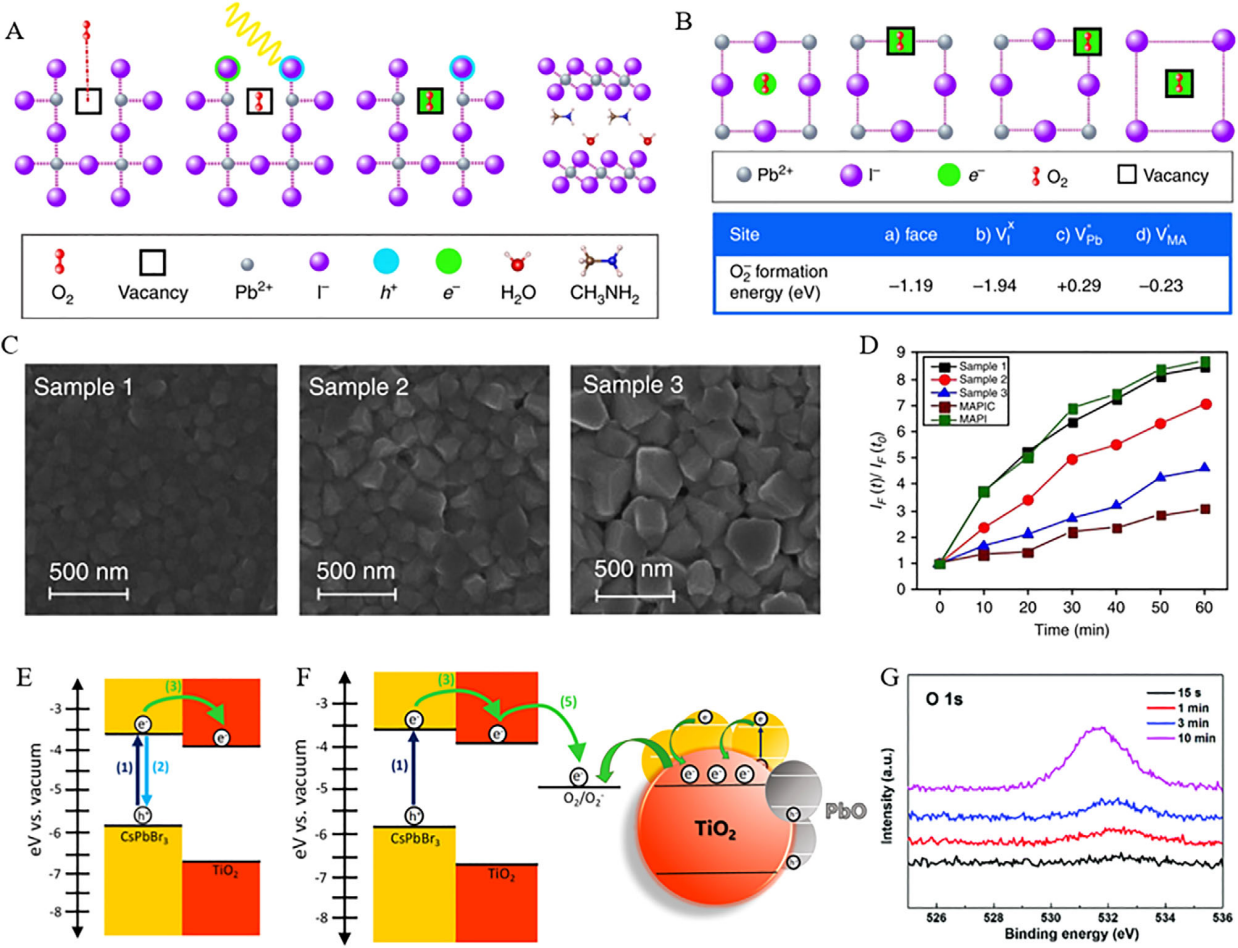
(A) Schematic illustrating O_2_ interaction with CH_3_NH_3_PbI_3_ in sequential steps. Reproduced with permission [24b]. Copyright 2017, Springer Nature. (B) Schematic depicting O_2_ binding and reduction sites in CH_3_NH_3_PbI_3_ ([001] plane) alongside corresponding superoxide formation energy. Reproduced with permission [24b]. Copyright 2017, Springer Nature. (C) Surface SEM images exhibiting crystal size variation in CH_3_NH_3_PbI_3_, categorized as small (sample 1), medium (sample 2), and large (sample 3). Reproduced with permission [24b]. Copyright 2017, Springer Nature. (D) Superoxide yield plotted against various crystal sizes of MAPbI_3_. Reproduced with permission [24b]. Copyright 2017, Springer Nature. (E) Energy level diagram illustrating the potential excited‐state pathways accessible to photogenerated charge carriers in CsPbBr_3_ nanocrystals adsorbed on TiO_2_ film. Reproduced with permission [45]. Copyright 2018, American Chemical Society. (F) Band diagram illustrating oxygen‐mediated electron transfer mechanism in ambient atmospheres, causing photodegradation of CsPbBr_3_ at TiO_2_ interfaces. Reproduced with permission [45]. Copyright 2018, American Chemical Society. (G) O1s spectra of CsPbI_2_Br films annealed at different times. Reproduced with permission [46]. Copyright 2019, Royal Society of Chemistry.

Apart from moisture, oxygen has long been another crucial factor that influences the preparation and stable operation of PSCs in ambient air. Generally, CH_3_NH_3_PbI_3_ undergoes rapid degradation in the oxygen environment, which was attributed to the formation of charge barrier ascribed to the reaction between MA^+^ and oxygen [42]. The potential mechanism for the formation of charge barriers is governed by the increased mobility of defects and electron trap density of the perovskite layer [43]. In this regard, Aristidou et al., [24b] reported the oxygen‐mediated degradation process of organic halide perovskites in light that might be facilitated by the photo‐excited electrons. It is obvious from Figure 4A that CH_3_NH_3_PbI_3_ reacts with oxygen to produce PbI_2_ in dark conditions (see Equation (6)) [24b]. Therefore, photoactive layer of CH_3_NH_3_PbI_3_ demonstrated an induced formation of superoxide species ($O_{2}^{-}$), whereas the active $O_{2}^{-}$‐species leading to the deprotonation of photo‐excited $CH_{3}NH_{3}{\mathrm{PbI}}_{3}^{*}$. This phenomenon may lead to the decomposition and formation of identical products as presented by Equation (7). Ultimately, iodine vacancies with a similar volume to $O_{2}^{-}$ forms a superoxide species as illustrated in Figure 4B, thus, oxygen molecules not only diffuse through grain boundaries but support the formation of superoxide species in these regions [24b].

(6) $$4CH_{3}NH_{3}\mathrm{Pb}I_{3}+O_{2}\to 4\mathrm{Pb}I_{2}+2I_{2}+2H_{2}O+4CH_{3}NH_{2}$$

(7) $$4CH_{3}NH_{3}{\mathrm{PbI}}_{3}^{*}+O_{2}^{-}\to 4\mathrm{Pb}I_{2}+2I_{2}+2H_{2}O+4CH_{3}NH_{2}$$

(8) $$4\mathrm{CsPb}X_{3}+\frac{1}{4}O_{2}\to \frac{1}{2}Cs_{2}O+\frac{1}{2}X_{2}+\mathrm{Pb}X_{2}$$

(9) $$4\mathrm{CsPb}X_{3}+\frac{1}{2}O_{2}\to \mathrm{CsX}+X_{2}+\mathrm{PbO}$$

(10) $$4\mathrm{CsPb}X_{3}+\frac{3}{4}O_{2}\to \frac{1}{2}Cs_{2}O+\frac{3}{2}X_{2}+\mathrm{PbO}$$

(11) $$\mathrm{CsPbB}r_{3}\left(h^{+}+e^{-}\right)+\mathrm{Ti}O_{2}\to \mathrm{CsPbB}r_{3}\left(h^{+}\right)+\mathrm{Ti}O_{2}\left(e^{-}\right)$$

(12) $$\begin{array}{ccc} & & \mathrm{CsPbB}r_{3}\left(h^{+}\right)+\mathrm{Ti}O_{2}\left(e^{-}\right)+O_{2}\\ & & \;\to \mathrm{CsPbB}r_{3}\left(h^{+}\right)+\mathrm{Ti}O_{2}+O_{2}^{-} \end{array}$$

From the above discussion, it can be understood that the photo degradation rate of perovskite materials is relatively close to their grain size. Thus, the rise in the quantity of grain boundaries led to a significant increase in the surface adsorption/reactive site density, as shown in Figure 4C,D, where CH_3_NH_3_PbI_3_ thin films with larger grains exhibited lower superoxide generation rates, while higher superoxide generation rates were attained with smaller grains [24b].

Inorganic perovskites with distinct chemical structures exhibit unique oxygen degradation as compared to the organic‐inorganic hybrid perovskites. Considering Equations (8)–(10), the oxidation products of CsPbX_3_ are directly correlated with oxygen content [44], where the interface electrons were easily transported between CsPbBr_3_ and metal oxides (TiO_2_, SnO_2_, and ZnO) [45]. For instance, the energy diagram in Figure 4E shows the electrons transfer from photoexcited CsPbBr_3_ to TiO_2_, while TiO_2_ being an excellent electron acceptor to form TiO_2_ ($e^{-}$) followed by Equation (11). In this aspect, electrons transfer from TiO_2_ to O_2_ in an oxygen atmosphere, thus leading to reduced oxygen ($O_{2}^{-}$) as followed by Equation (12). Thus, oxygen‐captured electrons resulted in the accumulation of holes in CsPbBr_3_ and further promoted the oxidative decomposition of perovskites (see Figure 4F) [45]. In addition, some other reports have demonstrated the chemically absorbed oxygen atoms on the surface of CsPbI_2_Br as presented in Figure 4G, which may lead to the generation of interstitial oxygen vacancies [46].

As discussed in Sections 2.1 and 2.2, we explored the degradation of perovskite materials, particularly by moisture and oxygen, which pose significant challenges to the stability and performance of PSCs. Moisture‐induced degradation involves complex phase transitions and structural alterations, leading to the formation of hydrated phases and ultimately irreversible degradation stages [29]. Moreover, oxygen‐mediated degradation processes contribute to the formation of charge barriers and the oxidative decomposition of perovskites, impacting their long‐term stability and performance [24b]. Despite these challenges, there are opportunities to leverage moisture and oxygen to improve the fabrication and quality of perovskite films. Controlled exposure to trace amounts of water can promote recrystallization and passivation of trap states, potentially enhancing film morphology and quality. Additionally, post‐treatment strategies involving oxygen exposure have been explored to reduce defect density in perovskite films. Therefore, optimizing fabrication conditions to balance the positive and negative effects of ambient conditions, such as humidity and oxygen content, can contribute to maximizing the quality and stability of perovskite films for PSCs applications.

## 3 Strategies of Enhanced Stability in PSCs

Although PSCs demonstrated enormous potential for the development of devices with high conversion efficiency mostly fabricated in inert atmosphere glove boxes, however, perovskite films have exhibited low coverage, numerous pinholes, and small grain sizes prepared in ambient air. These perovskite films possess high sensitivity to environmental factors, that is, moisture, oxygen, and light may crucially impede their commercialization. Therefore, some popular strategies are summarized in the below section (Table 1) to explore the stable PSCs with the following aspects of composition, additive, solvent, and interface engineering, respectively.

**TABLE 1:** Summary of strategies to enhance perovskite stability.

Strategy		Device structure	RH [%]	PCE [%]	Ref.
Compositional engineering	Mixed‐cation and/or mixed‐halide	FTO/TiO_2_/FA_1−_ *_x_* Cs*_x_* PbI_3_/Spiro‐OMeTAD/Ag	15	16.1	[49]
		FTO/TiO_2_/FA_1−_ *_x_* Cs*_x_* PbI_3_/Spiro‐OMeTAD/Au	55	16.5	[50]
		FTO/TiO_2_/Cs*_x_* (FA_0.83_Cs_0.17_)_1−_ *_x_* Pb(I_0.83_Br_0.17_)_3_/Spiro‐OMeTAD/Au	15–25	20.8	[53]
		FTO/TiO_2_/(FAPbI3)_1−_ *_x_* (MDA, Cs)*_x_*/Spiro‐OMeTAD/Au	25	24.4	[54]
		FTO/TiO_2_/MAPbI_3‐_ *_x_* Cl*_x_*/Spiro‐OMeTAD/Au	15	14.15	[56]
		FTO/TiO_2_/CsFAMAPbIBr/Spiro‐OMeTAD/Au	40–50	19.7	[61]
		ITO/SnO_2_/CsPbI_2.25_Br_0.75_/PTAA/MoO_3_/Ag	40–50	17.08	[60]
		FTO/TiO_2_/MAPb(SCN)_2_I/Spiro‐OMeTAD /Au	95	8.3	[62]
		FTO/SnO_2_/Perovskite/PEAI/Spiro‐OMeTAD /Au	20–60	24.16	[67]
		FTO/c‐TiO_2_/FA_0.98_MA_0.02_PbI_3_/o‐F‐PEAI/Spiro‐OMeTAD/Au	30–85	25.08	[63]
Additive engineering	ILs	FTO/c‐TiO_2_/mp‐TiO_2_/mp‐ZiO_2_/mp‐Carbon/MAPbI_3_:MAAc	35	13.54	[71]
		FTO/TiO_2_/MAPbI_3_:[bvbim]Cl/Spiro‐OMeTAD /Au FTO/TiO_2_/FAMAPbI_3_:[bvbim]Cl/Spiro‐OMeTAD /Au	30 50	17.18 19.92	[72]
		ITO/PTAA/Perovskite:BAAc/C_60_/BCP/Ag	35	20.1	[74]
		ITO/PEDOT:PSS/perovskite:MAFa/Spiro‐OMeTAD/Au	50	24.09	[75]
		ITO/SnO_2_/Perovskite:TEAPF_6_/PCBM/Ag	40	22.13	[76]
		ITO/Me‐4PACz/perovskite:DMAFo/PDADI/C_60_/Ag	35–50	24.72	[77]
	Ammonium salt	FTO/TiO_2_/perovskite:MACl/spiro‐OMeTAD/Au	30	21.65	[78]
		ITO/PTAA/perovskite:MACl/C_60_/BCP/Ag	20–40	23.1	[79]
		FTO/c‐TiO_2_/mp‐TiO_2_/perovskite:MACl/PEAI/Spiro‐OMeTAD /Au	25	23.48	[80]
	Polymer	FTO/TiO_2_/ CsPbI_3_:PHPS/PEAI/Spiro‐OMeTAD /Au	60	19.17	[81]
		ITO/NiO*_x_*/MAPbI_3_:PAA/PCBM/BCP/Ag	50	20.29	[82]
		ITO/SnO_2_/Perovskite:PAB/PM6‐T10/MoO_3_/Ag	50	21.13	[83]
	Small organic molecules	ITO/PEDOT:PSS/MAPbI_3_:C_60_‐Ta/PCBM/BPhen/Ag	25–50	16.46	[87]
		ITO/PEDOT:PSS/MAPbI_3_:C_60_/PCBM/BPhen/Ag ITO/PEDOT:PSS/MAPbI_3_:PCBM/PCBM/BPhen/Ag	25–50 25–50	16.59 15.94	[87]
		ITO/SnO_2_/FA_0.65_MA_0.35_PbI_3‐_ *_x_* Cl*_x_*:PC61B‐TEG/Spiro‐OMeTAD /Au	25	23.34	[84]
		FTO/TiO_2_/MAPbI_3_:MP/PEAI/Spiro‐OMeTAD /Au FTO/TiO_2_/MAPbI_3_:MP/PEAI/Spiro‐OMeTAD /Au FTO/TiO_2_/MAPbI_3_:PY/PEAI/Spiro‐OMeTAD /Au	50 50 50	17.03 15.49 14.34	[88]
		FTO/TiO_2_/MAPbI_3_:Zn‐TTB/PEAI/Spiro‐OMeTAD /Au	40	23.14	[85]
Solvent engineering	ACN	FTO/TiO_2_/C_60_/MAPbI_3_/spiro‐OMeTAD/Au		19.0	[90]
	ACN	FTO/SnO_2_/MAPbI_3_/spiro‐OMeTAD/Au		17.82	[91]
	PEACL	FTO/PTAA/MAPbI_3_/C_60_/BCP/Cu	30	20.05	[92]
	2ME	ITO/PTAA/ MAPbI_3_/C_60_/BCP/Cu	50	19.16	[93]
	Pb(NO_3_)_2_/H_2_O	FTO/TiO_2_/MAPbI_3_/spiro‐OMeTAD/Au	30	12.58	[94]
	Pb(NO_3_)_2_/H_2_O	FTO/c‐TiO_2_/mp‐TiO_2_/perovskite/spiro‐OMeTAD/Au	15	23.74	[97]
	IPA_100_	ITO/NiO*_x_*/MAPbI_3_/PC_61_BM/Ag	50–60	16.27	[98]
	RNH_3_Cl	FTO/SnO_2_/FAPbI_3_/spiro‐OMeTAD/Au	20	25.1	[99]
	CTAC	ITO/SnO_2_/perovskite/spiro‐OMeTAD/Au	30	23.4	[103]
	EA	FTO/SnO_2_/MAPbI_3_/spiro‐OMeTAD/Au	37.5	17.83	[105]
	EA	ITO/SnO_2_/MAPbI_3_/spiro‐OMeTAD/Au	20–50	20.11	[100]
	DMSO and DE	FTO/c‐TiO_2_/mp‐TiO_2_/SnO_2_/MA_0.5_FA_0.5_PbI_3_/Spiro‐OMeTAD/Au		21.8	[106]
	DMSO and DE	FTO/SnO_2_/(FAPbI_3_)0.875(CsPbBr3)_0.125_/Spiro‐OMeTAD/Au	0.3	23.02	[101]
Interface engineering		FTO/TiO_2_/MAPbI_3−_ *_x_* Cl*_x_*/Chl‐1/P3HT/Ag	25–35	11.44	[109]
		FTO/c‐TiO_2_/mp‐TiO_2_/MAPbI_3_/DNA‐CTMA/Spiro‐OMeTAD/Au	50	20.63	[110]
		ITO/SnO_2_/(FAPbI_3_)*_x_* (MAPbBr_3_)_1−_ *_x_*/theophylline/Spiro‐OMeTAD/Ag	40	22.6	[111]
		FTO/c‐TiO_2_/mp‐TiO_2_/FA_0.65_MA_0.35_PbI_3−x_Cl_x_/K‐type M13/ Spiro‐OMeTAD/Au		23.6	[112]
		FTO/TiO_2_/CsPbI_3_ QDs/spiro‐OMeTAD/Au	20	11.2	[113]
		ITO/PEDOT:PSS/Perovskite/PCBM/BCP/Ag		15.46	[114]
		ITO/SnO_2_/(FAPbI_3_)_1−_ *_x_* (MAPbBr_3_)*_x_*/PEAI/PTAA/Au	30–40	23.32	[115]
		ITO/SnO_2_/Rb_0.02_(FA_0.95_Cs_0.05_)_0.98_PbI_2.91_Br_0.03_Cl_0.06_/MTDAA/spiro‐OMeTAD /Au	10–20	21.92	[107]
		ITO/SnO_2_/Rb_0.02_(FA_0.95_Cs_0.05_)_0.98_PbI_2.91_Br_0.03_Cl_0.06_/ADAA/spiro‐OMeTAD /Au	20–30	23.18	[116]
		ITO/SnO_2_/FA_0.85_MA_0.15_PbI_3_/DDPUD/spiro‐OMeTAD /Au	10–20	24.47	[108]

Abbreviations: [bvbim]Cl, 1,3‐bis(4‐vinylbenzyl)imidazolium chloride; 2ME, 2‐methoxyethanol; ACN, acetonitrile; ADAA, 1‐adamantaneacetic acid; BAAc, butylammonium acetate; BCP, bathocuproine; CTAC, cetyltrimethylammonium chloride; DDPUD, ulose‐3,5‐dibenzoate; DE, diethyl ether; DMAFo, dimethylammonium formate; EA, ethyl acetate; IPA_100_, isopropanol(99.5%); K‐type M13, Lysine modified M13;MAAc, methylammonium acetate; MAFa, methylammoniumformate; MAFa, methylammoniumformate; Me‐4PACz, (4‐(3,6‐dimethyl‐9H‐carbazol‐9‐yl)butyl)phosphonic acid PDADI, 1,3‐propanediamine dihydroiodide; MTDAA, (5‐mercapto‐1,3,4‐thiadiazol‐2‐ylthio)acetic acid; PAA, poly(acrylic acid); PAB, phenolic hydroxyl‐substituted polyamide derivative; PCBM, (6,6)‐phenyl‐C_61_‐butyric acid methyl ester; PEACl, phenethylammonium chloride; PEAI, phenethylammonium iodide; PEAI, phenylethylammonium iodide; PHPS, polymer perhydropolysilazane; RNH_3_Cl, alkylammonium chloride; TBP, 4‐tert‐butyl pyridine; TEAPF_6_, tetraethylammonium hexafluorophosphate; TTB, 1‐(triazol‐1‐ly)‐4‐tetrazol‐5‐ylmethyl) benzene.

### 3.1 Composition Engineering

**FIGURE 5:**
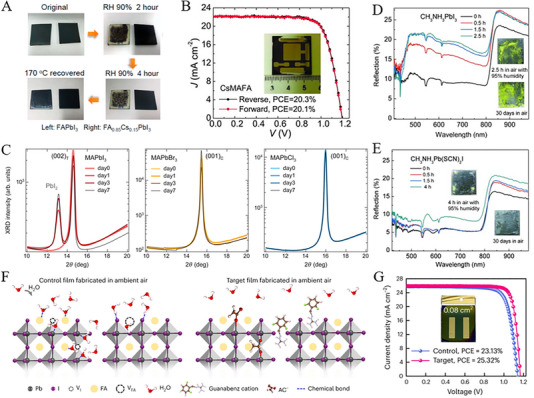
(A) Images of thin films of FAPbI_3_ and FA_0.85_Cs_0.15_PbI_3_ exposed to high‐humidity conditions. Reproduced with permission [49]. Copyright 2016, American Chemical Society. (B) Current–voltage (*J*‐*V*) characteristics of the most efficient large‐area (1.1 cm^2^) CsMAFA perovskite solar cell. Reproduced with permission [52]. Copyright 2017, The American Association for the Advancement of Science. (C) Evolution over time of the main XRD peak of the perovskite phase for MAPbI_3_, MAPbBr_3_, and MAPbCl_3_. Reproduced with permission [55]. Copyright 2015, American Chemical Society. Accelerated stability tests performed on (D) MAPbI_3_ and (E) MAPb(SCN)_2_I exposed to air with 95% RH. Reproduced with permission [62]. Copyright 2015, John Wiley and Sons. (F) Schematic depiction of moisture‐induced decomposition and the reduction in moisture sensitivity resulting from the inclusion of GBA. Reproduced with permission [63]. Copyright 2023, Springer Nature. (G) *J*‐*V* curves of the leading control and target PSCs with an aperture area of 0.08 cm^2^. Reproduced with permission [63]. Copyright 2023, Springer Nature.

In organic–inorganic hybrid halide perovskites, the A cations (MA^+^, FA^+^, and Cs^+^) are positioned at the center of the three‐dimensional perovskite structure, playing a crucial role in determining its structure and size, which directly affects the material's stability and optoelectronic properties [47]. Similarly, the X anions (e.g., halides, pseudohalides, and tetrafluoroborate salts) allocated at the six vertices of perovskite octahedral BX_6_ structure, indicating the potential replacement of X‐site anions (I) with alternate anions. This substitution can modify the structure from tetrahedral to cubic, thereby affecting bandgap, crystal structure, and charge transport [47]. In addition, reports revealed a dual‐cation MA/FA mixture, and the formation of α phase FAPbI_3_ was attained by introducing a small amount of MA, resulting in a mixture exhibiting superior thermal stability and structural stability with PCE over 20% [48]. In contrast, the inherent thermal and chemical instability of organic components, as well as phase transitions, lead to inferior performance of PSCs, therefore solid‐state alloying was proposed to improve the tolerance factors and stability of perovskite films. For instance, mixed‐cations as FA_1−_ *_x_* Cs*_x_* PbI_3_ compound with tolerance factor of unity were encountered, and thus the stability of photoactive α phase enlarged with optimal PCE of 16% [49]. Interestingly, FA_0.85_Cs_0.15_PbI_3_ perovskite films exhibit impressive humidity stability at 90% RH as compared to pure FAPbI_3_ films (see Figure 5A). Similarly, a mixed‐cation perovskite FA_1−_ *_x_* Cs*_x_* PbI_3_ was reported by Lee et al. [50], with an increased average PCE of 16.5%, and suggest the enhancement in optical and moisture stability were majorly facilitated by FA^+^ and iodide ions interactions [51]. Therefore, the introduction of Cs into FA/MA may improve the crystal growth and morphology of perovskites, resulting in effective charge collection [52]. PSCs‐based FA/MA/Cs cations demonstrated a PCE of over 20% with stable performance in ambient air (see Figure 5B). However, the preparation of triple‐cation perovskites through one‐step solution deposition and low‐temperature annealing under humidity conditions resulted in a PCE of 20.8% [53]. It can be noted that alloyed cations may influence the bandgap, carrier dynamics, and stability of the perovskite, while introducing lattice strain may generate some undesired carrier trap sites. For this reason, Kim et al., [54] incorporated 0.03 mol fraction of both methylenediammonium (MDA) and Cs cations, leading to increased carrier lifetime with reduced Urbach energy and lower defect concentration.

Halogen elements chlorine and bromine of the same group exhibit competitive properties of prepared MAPbBr_3_ and MAPbCl_3_ films [55]. For instance, Figure 5C demonstrates the diffraction pattern of MAPbI_3_ with some obvious peaks of PbI_2_ and indicates the decomposition of MAPbI_3_ into PbI_2_. Whereas, a negligible decomposition in MAPbBr_3_ crystal was observed with unchanged diffraction peaks of MAPbCl_3_. This feature can be attributed to a decrease in the halide ion radius (*r*_I_ > *r*_Br_ > *r*_Cl_), and thus resulted in an increased bandgap of MAPbI_3_, MAPbBr_3_, and MAPbCl_3_. Mixed halide perovskites due to the substituted $I^{-}$ by $Br^{-}$ or $Cl^{-}$ has significantly enhanced the stability of perovskites without sacrificing their performance by well‐controlled composition. In this aspect, Dharani et al., [56] achieved an optimal PCE of 14.15% in MAPbI_0.9_Cl_0.1_ films prepared through sequential deposition with the addition of halides. In fact, a minute quantity of Cl incorporated into MAPbI_3_ turned the phase transition from an unstable tetragonal to a stable cubic phase at room temperature. Similarly, Xiao et al. [57]. elucidated the Cl‐assisted crystallization pathway of perovskite within mesoporous metal oxides, revealing the effective role of $Cl^{-}$ ions in inhibiting the formation of intermediate MA_2_Pb_3_I_8_·2DMSO. This Cl‐assisted approach boosted the photoelectric properties and achieved an elevated efficiency ranging from 12.23% to 16.85% comparatively to pristine MAPbI_3_ films. In addition to this discussion, mixed halide perovskites incorporated with bromine and iodine (Br−I) might be crucial for engineering wide‐bandgap PSCs; however, the increased content of $Br^{-}$ frequently leads to extra defect states. In this regard, a reduced defect density was attained by Jiang et al., [58] through a rapid crystallization of $Br^{-}$ and gas quenching, however, a record efficiency of ≈17.46% makes CsPbI_2_Br PSCs more promising through air processing [59]. Zhang et al., [60] employed an additive‐assisted airflow drying (AAD) technique and successfully fabricated the mixed halide inorganic PSCs, however, the combined airflow drying with PbAc_2_·3 H_2_O additive under ambient air conditions was reported for inorganic perovskite films. The AAD method exhibited larger grains in various films that govern higher crystallinity and fewer defects as compared to the control films. For example, the incorporated CsPbI_2.25_Br_0.75_ solar cells with AAD demonstrated a remarkable PCE of 18.49% under ≈50% RH. On the other hand, Lee et al., [61] proposed a two‐step spin‐coating and static spin‐coating method of mixed‐cation perovskites in CsFAMAPbIBr (40–50% RH), and hence, distinct Ostwald ripening and ion exchange behaviors lead to the variant chemical and morphological properties in the corresponding films. In addition, perovskites prepared by dynamic spin‐coating exhibited a residual PbI_2_ with a uniform surface, though less residual PbI_2_ and a nonuniform surface were observed in static spin‐coating. Likewise, the planar solar cells based on dynamically coated CsFAMAPbIBr perovskites demonstrated a superior PCE of ≈19.70% as compared to PCE of 16.01% by statically coated CsFAMAPbIBr.

Pseudo‐halide anions exhibit tremendous properties compared to halogen anions and reveal enhanced film crystallinity. Thiocyanate (SCN^−^), tetrafluoroborate (${\mathrm{BF}}_{4}^{-}$), and bis(trifluoromethanesulfonyl)imide (TFSI^−^) ions emerge as effective substitutes for halogen ions due to their optimized band gap, crystallinity, and defect density. In this scenario, Chen et al., [64] prepared MAPbI_1−_ *_x_* (SCN)*_x_* films through a simple two‐step method composed of spin‐coating mixture of PbI_2_ and Pb(SCN)_2_ followed by MAI layer. The resulting MAPbI_1−_ *_x_* (SCN)*_x_* films exhibited larger crystal sizes and fewer charge traps with an optimum device efficiency of ≈11.07%. Compared to the conventional MAPbI_3_ perovskites by PbI_2_, unencapsulated MAPbI_1−_ *_x_* (SCN)*_x_* film devices demonstrate favorable reproducibility and stability owed by the incorporated SCN^−^. These results revealed a decrease of PCE about 8% in MAPbI_1−_ *_x_* (SCN)*_x_* perovskite, however, MAPbI_3_ perovskite decreased the PCE up to 42% after 1 h in light. These results indicate that the addition of a small amount of SCN^−^ in MAPbI_3_ has significantly reduced the photo‐induced degradation. Thus, Jiang et al., [62] substituted two SCN^−^ in MAPbI_3_, and a degradation of MAPbI_3_ film was noted in an air environment after 1.5 h, whereas the MAPb(SCN)_2_I film exhibited no significant degradation even after 4 h (see Figure 5D,E). The formation constant of lead halide serves as an indicator of the binding affinity between halides and central Pb^2+^. Some theoretical studies have reported the formation constants of 3.5 and 7 for ${\mathrm{PbI}}_{4}^{2-}$ (in MAPbI_3_) and $\mathrm{Pb}{\left(\mathrm{SCN}\right)}_{4}^{2-}$ (in MAPb(SCN)_2_I) respectively, suggesting a highly stable structure of MAPb(SCN)_2_I [65]. This phenomenon may be attributed to the linear‐shaped SCN^−^ with pairs of electrons, thus possible strong interactions with Pb^2+^ facilitate strong electrostatic forces [62]. Besides, Zhang et al., [66] utilized tetrafluoroborate (${\mathrm{BF}}_{4}^{-}$) anion in (FAPbI_3_)_0.83_(MAPbBr_3_)_0.17_ perovskite films for the first time and a remarkable PCE exceeding 20.16% was attained. Incorporating ${\mathrm{BF}}_{4}^{-}$ into the mixed‐ion perovskite crystal lattice induced lattice relaxation, prolonged photoluminescence lifetime, heightened recombination resistance, and significantly reduced trap density in both perovskite films and derived solar cells. Therefore, Tian et al., [67] proposed an anion stabilization strategy to enhance the durability of perovskite photovoltaics during a two‐step air‐processed procedure. This approach has opened the search for interionic bonding on ink properties, film crystallization, photovoltaic performance, etc. Additionally, the incorporation of pseudohalide TFSI^−^ results in polyhalides and organic salt inks while promoting a uniform composition and controlled crystal growth of subsequent perovskite layers. As a result, the optimized PSCs have achieved an impressive efficiency of 24.16%, demonstrating long‐term stability and minimal leakage. This effort may cause mitigated cation vacancies by organic groups with suitable molecular sizes according to formamidinium ions (FA^+^). Moreover, pseudohalide anions such as HCOO− and CH_3_COO− with high electronegativities not only suppress the anion‐vacancy defects but also facilitate the film's crystallinity. For instance, Yan et al. [63], have addressed the cation and anion vacancies in guanabenz acetate salt (GBA) as shown in Figure 5F, where the perovskite hydration is inhibited in ambient air and hence an impressive PCE of 25.32% (see Figure 5G).

In conclusion, the manipulation of cations (a) and anions (X) in hybrid halide perovskites through composition engineering is pivotal for enhancing the stability and performance of PSCs. Mixed‐cation formulations such as FA/MA/Cs have shown promise in improving thermal stability, with some formulations achieving PCEs exceeding 20%. Incorporating halogen elements (Cl, Br) or pseudohalides (SCN^−^, ${\mathrm{BF}}_{4}^{-}$, TFSI^−^) has been found to enhance stability and crystallinity, with mixed halide perovskites demonstrating improved stability without compromising performance. Strategies aimed at anion stabilization, such as the incorporation of ${\mathrm{BF}}_{4}^{-}$, have resulted in enhanced PCE and long‐term stability [62]. Pseudohalide anions like TFSI^−^ have facilitated uniform composition and controlled crystal growth, leading to remarkable efficiencies reaching 24.16% [67]. Moreover, the utilization of high electronegativity pseudohalides has been effective in suppressing defects and enhancing crystallinity, as evidenced by GBA, which achieved an impressive PCE of 25.32% [63]. Overall, composition engineering strategies offer promising avenues for advancing the stability and efficiency of PSCs.

### 3.2 Additive Engineering

Additive engineering primarily involves the introduction of additives to improve the crystallization of perovskite's film, defects, and regulated interface [68]. Thus, additives can effectively promote the homogeneous nucleation or crystallization kinetics of perovskites. In brief, controlling the nucleation and crystal growth is crucial for achieving a good surface coverage and desired crystal size and may lead to reduced electrical shunt, recombination, and trap states [69].

**FIGURE 6:**
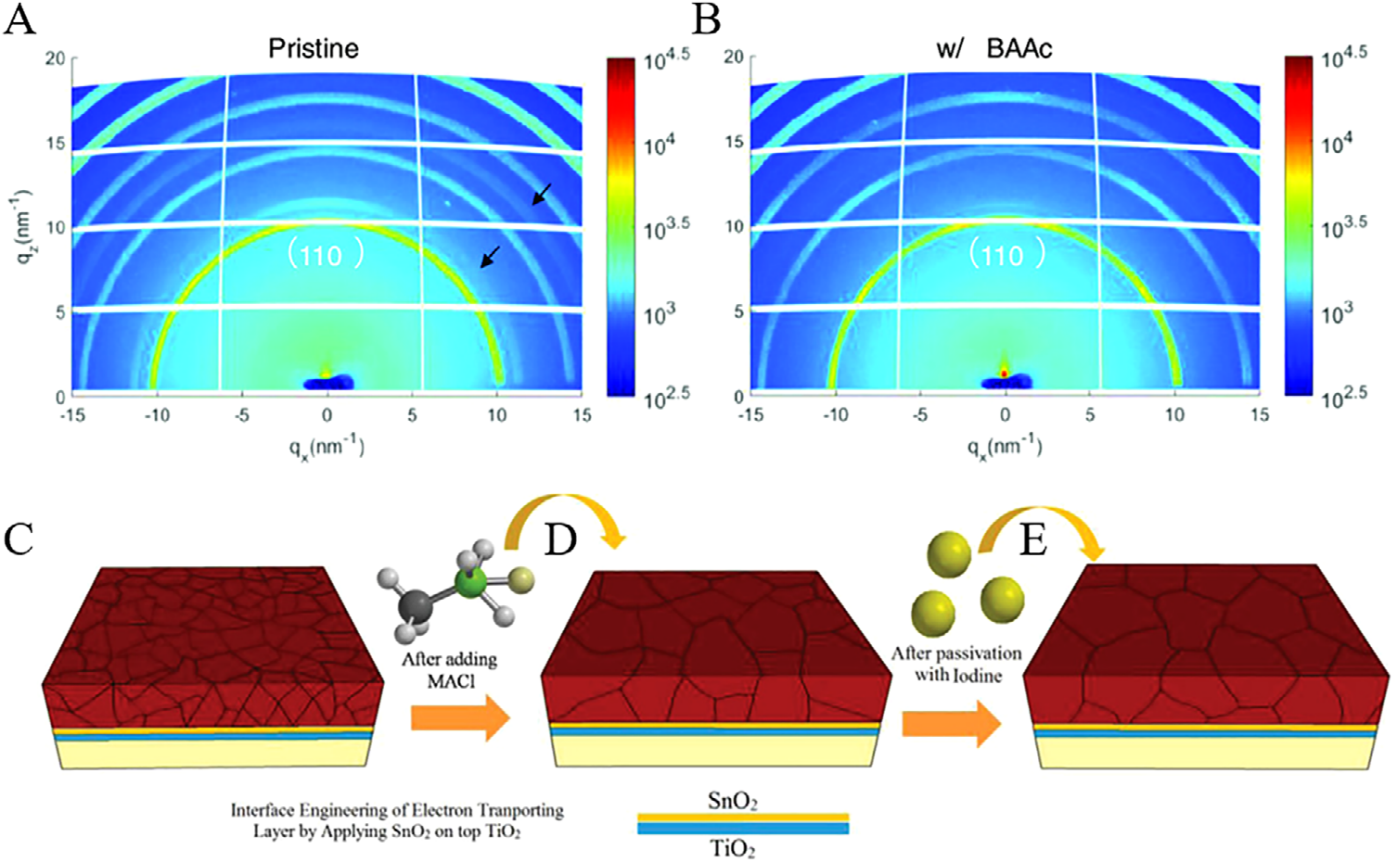
2D GIWAXS images of (A) the pristine and (B) the BAAc‐treated perovskite film. Reproduced with permission [74]. Copyright 2022, John Wiley and Sons. The diffraction rings at approximately 11 and 16 nm^−1^ in (A) are indicated by black arrows. Schematics illustrating the triple A‐cation perovskite films (C) without and (D) with MACl additive, and (E) after passivation with iodine. Reproduced with permission [78]. Copyright 2018, John Wiley and Sons.

Ionic liquids (ILs) belong to the category of salts, composed of organic cations (e.g., pyridinium, piperidinium, imidazolium, and pyrrolidinium) and organic/inorganic anions (e.g., halides, phosphates, sulfonates, and imides) [70]. Instead of utilizing the traditional high‐toxic solvents such as *N*, *N*‐dimethylformamide (DMF) and dimethyl sulfoxide (DMSO), protonated amine carboxylic acid ILs have emerged as substitute precursor solutions for PSCs [5c]. In this regard, Wang et al., [71] introduced methylammonium acetate (MAAc) as a liquid additive in MAPbI_3_ perovskite and hence fabricated high‐performance carbon‐based mesoscopic perovskite solar cells (MPSCs). Through systematic investigation, MAAc was preferentially founded to incorporate with PbI_2_ to realize MAPbI_3−_ *_x_* (AC)*_x_* the intermediate phase, thereby effectively regulating MAPbI_3_ crystallization in the triple‐mesoscopic layer. Furthermore, the optimized MAAc‐engineered MPSCs exhibited enhanced crystallinity, reduced defect density, and improved pore filling, which effectively resulted in a champion PCE of 13.54%. Likewise, Xia et al., [72] presented a novel in situ polymerizable ionic liquid of 1,3‐bis(4‐vinylbenzyl)imidazolium chloride ([bvbim]Cl) and fabricated a perovskite film in humid conditions, achieving high‐quality and long‐term stability. Therefore, the unencapsulated MAAc‐engineered MPSCs demonstrated superior air stability with PCE of 90% even after 50 days of storage in the dark. Considering the high boiling point and notable hydrophobic properties, ILs are capable of modulating the wetting behavior of precursors, and thus nucleation barrier of perovskites can be optimized. On the other hand, the hydrogen bonds with perovskites facilitate a homogeneous nucleation [73]. This effect can be referred to Ran et al., work as IL and butylammonium acetate (BAAc) were utilized to modulate the crystallization and hence a stable PSCs with a high efficiency of 20% was attained [74]. The interaction between BAAc molecules and halide is established through C═O─Pb chelate bonds and N─H⋯I hydrogen bonds, and thus the suppression of crystal growth in perovskite film may be controlled. Additionally, GIWAXS analysis (see Figure 6A,B) has provided further evidence that elucidates the oriented crystallization, which can be attributed to the bonding interactions between BAAc and [PbI_6_]^4−^ framework. This approach was further realized by Wang et al. [75], in methylammoniumformate (MAFa) ILs, and the formation of perovskite films were attained in PbI_2_. This phenomenon suggests the chemical interaction of ILs additive in perovskite precursor solution and led to the infiltration of organic salts. For example, Guo et al., [76] incorporated pseudohalide‐based ionic tetraethylammonium hexafluorophosphate (TEAPF_6_) into perovskite films, and demonstrated some governed multiple effects such as; ${\mathrm{PF}}_{6}^{-}$ anions filled halide vacancies, suppressed ion migration, and defects of organic cations. Additionally, TEAPF_6_ imparted hydrophobicity to the perovskite films, leading to improved stability and achieving a notable PCE of 22.13% in the devices. Moreover, Meng et al., [77] introduced a “whole‐process” stabilizer with a protic ionic liquid, dimethylammonium formate (DMAFo), facilitating the fabrication of p–i–n solar cells under ambient air conditions while achieving a stabilized efficiency of 24.72%. The reducing properties of DMAFo play a pivotal role in impeding the deprotonation of organic cations and the oxidation of iodide ions, thereby ensuring prolonged storage of the perovskite solution in ambient air, even under elevated temperatures. This protective mechanism extends throughout the transition from solution to solid state, enhancing the crystallinity of the perovskite and mitigating energetic disorder and atomic defects (notably metallic lead and halide vacancies) for non‐radiative recombination.

Efforts have been made to incorporate ammonium salt derivatives as additives in perovskite precursor solutions, realizing impressive crystalline properties and surface morphology. Notably, Tavakoli et al., [78] demonstrated the suppression of defects by incorporating MACl into precursor solution, yielding high‐quality triple‐cation perovskite films. Additionally, engineering the perovskite interface with electron and hole through a material facilitated efficient electron extraction, consequently mitigating carriers recombination in planar SnO_2_/TiO_2_ double layer oxide and iodine passivation (see Figure 6C–E). These efforts culminated in a great enhancement of the solar‐to‐electric change efficiency of 21.65% with photovoltage ($V_{\mathrm{oc}}$) of ≈1.24 V. In addition, Bi et al., [79] revealed a distinct role in volatile additives (NH_4_Cl, FACl, and MACl) and MA‐based additives (MACl, MABr, and MAI), suggesting the crystallization pathways of perovskite. In this regard, non‐MA volatile additives (NH_4_Cl and FACl) have facilitated the crystallization and gradually lowered the phase transition temperature, which may rapidly induce the formation of MA‐rich nuclei. Moreover, volatile MACl significantly promoted secondary crystallization growth during annealing, leading to optimized PSCs with a PCE of 23.1%. Similarly, Kim et al., [80] investigated that MACl additive plays a key role in FAPbI_3_‐based perovskite, however, MACl not only encouraged large grain size but stabilized the intermediate phase that ultimately achieved a PCE ≈23.48%.

**FIGURE 7:**
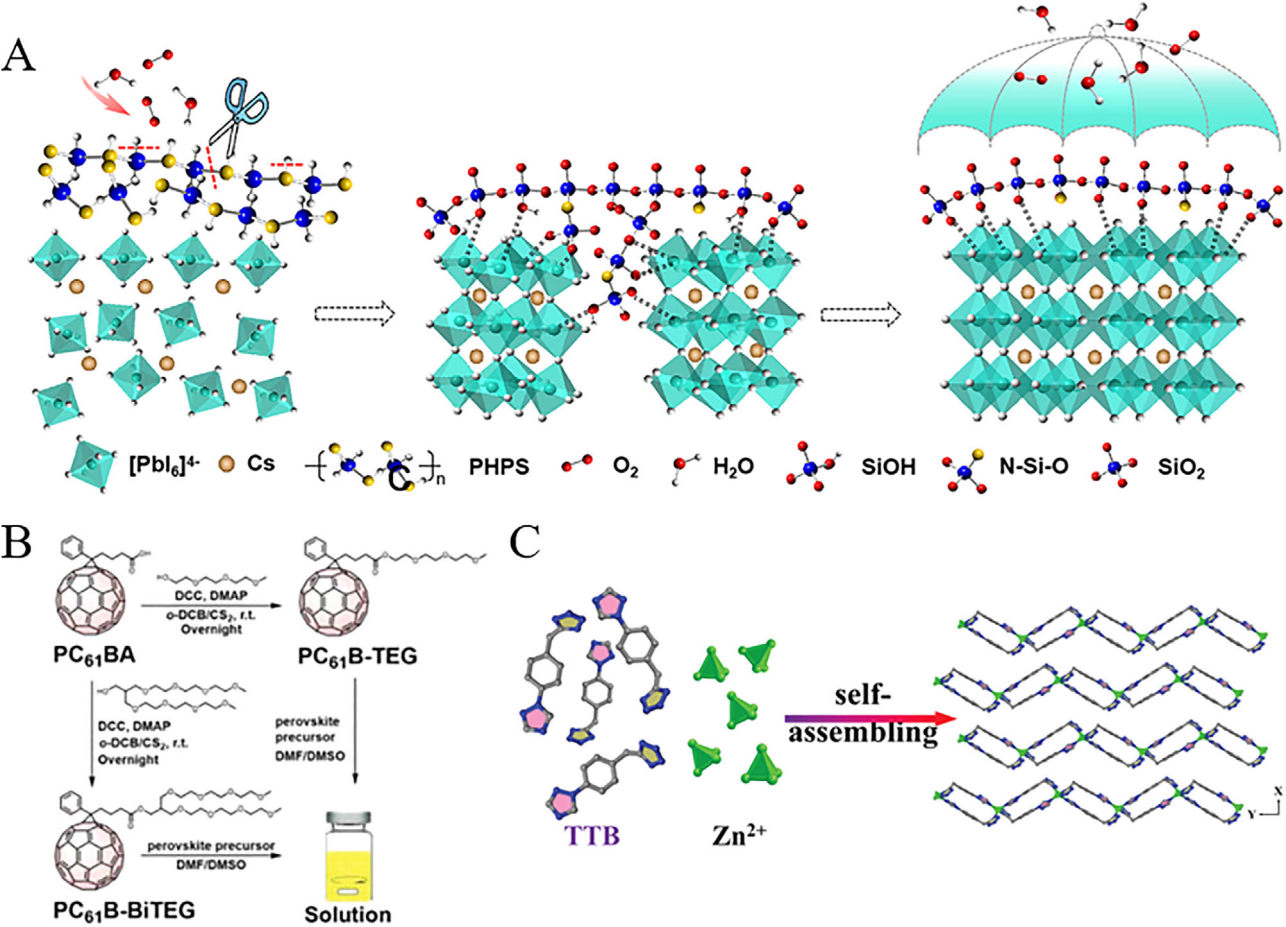
(A) Schematic representation of the growth process for CsPbI_3_ treated with PHPS under ambient conditions. Reproduced with permission [81]. Copyright 2023, John Wiley and Sons. (B) Illustration depicting the synthesis and blending process of PC_61_B‐TEG and PC_61_B‐BiTEG. Reproduced with permission [84]. Copyright 2022, John Wiley and Sons. (C) The synthesis process and *z*‐axis view of 1D crystal structures of Zn‐TTB are depicted schematically. Reproduced with permission [85]. Copyright 2022, John Wiley and Sons.

Polymers, high‐molecular‐weight compounds ranging from 10^4^ to 10^6^ g/mol, consist of several identical and interconnected structures with covalent bonds. They have been extensively utilized in additive engineering according to their distinctive functional groups, that is, the novel curing‐anti‐solvent method for perhydropolysilazane (PHPS) incorporated into methyl acetate produced a stable black‐phase CsPbI_3_ perovskite films under ambient air conditions [81]. The schematic illustration for PHPS treated CsPbI_3_ (see Figure 7A) presents the cross‐linked PHPS mitigated moisture erosion and a hydrolyzate silanol network (–Si(OH)_4_–) that may regulate the growth of perovskite crystal. Meanwhile, the polycondensation adduct between Si–O–Si/Si–O–Pb strongly binds with CsPbI_3_ grains and serves as a protective layer to impede the phase transition. As a result, the optimized addition of PHPS in an anti‐solvent solution resulted in the PCE 16.22% to 19.17% of CsPbI_3_‐based solar cells. Furthermore, Li et al., [82] employed three passivation molecules; poly(vinyl alcohol) (PVA), polymethyl acrylate (PMA), and poly(acrylic acid) (PAA), to analyze the passivation effect in ─OH, ─C═O, and ─COOH groups. Specifically, PVA (─OH) forms hydrogen bonds with perovskites, PMA (─C═O) complexes with uncoordinated Pb^2+^, while PAA (─COOH) selectively interacts with MA^+^ and I^−^ ions through hydrogen bonding. Consequently, the incorporated devices PAA‐MAPbI_3_ realized a champion efficiency of 20.29% with an open‐circuit voltage of ≈1.13 V, which can be attributed to the reduced carrier nonradiative recombination. Additionally, flexible PAA‐incorporated PSCs with a cross‐linked network exhibited remarkable flexural endurance and thus retained PCE of 80% after 3000 bending cycles. Li et al., [83] used phenolic hydroxyl‐substituted polyamide derivative (PAB) as a polymer additive in perovskite active layers, where the ─OH and ─COOH groups serve as Lewis bases. These passivation groups could be connected through a flexible polymer that effectively passivates the trap states at perovskite surface and grain boundaries.

Besides polymers, small organic molecules are ideal as perovskite precursor additives for PSCs due to their compact size, which facilitates penetration into perovskite lattice for effective passivation. Commonly employed small organic molecules in PSCs additive engineering include fullerene derivatives, pyridine, thiourea, and thiazole [86]. Hu et al., [87] systematically compared fullerene‐derivative additives as C_60_, (6,6)‐phenyl‐C_61_‐butyric acid methyl ester (PCBM), and C_60_‐Taurine, and found a slightly lower PCE of PSCs incorporating C_60_‐Taurine (16.46%) than that of C_60_ (16.59%) in contrast PCBM (15.94%). The above efforts conclude that the bare C_60_ cage primarily accelerates the extraction of electrons, while side chains on C_60_ enhance the stability of PSCs by reducing defect states. In this regard, Kim et al., [84] addressed the challenging immiscibility of fullerene derivatives and perovskite precursors by the synthesis of PC_61_B‐TEG and PC_61_B‐BiTEG (see Figure 7B). The high solubility of these derivatives in polar solvents may facilitate the gradual permeation of the fullerene additives into the perovskite film, and hence efficient passivation of perovskite defects with ultimate device efficiency of 23.34%. Further, Zhang et al., [88] introduced three pyridine molecules (pyridine (PY), 4‐methyl‐pyridine (MP), and 4‐tert‐butyl pyridine (TBP)) into perovskite layers, where pyridine molecules exhibited varied electron‐pair‐donor abilities (TBP > MP > PY) due to their differences in molecular structure. This phenomenon resulted in enhanced defect passivation with increased functional group‐Pb^2+^ interactions. However, the hydrophobic nature of these molecules may improve the moisture stability of devices, enhancing crystal quality and light harvesting ability by a slower growth process. Thus, PSCs incorporated TBP, MP, and PY demonstrated the PCEs of 17.03%, 15.49%, and 14.34%, respectively. Besides, Wang et al., [85] systematically utilized metal‐organic frameworks (MOFs) to enhance the stability and structural arrangement of an organic small molecule, resulting in a remarkable PCE of 23.14%. Specifically, ligand 1‐(triazol‐1‐ly)‐4‐tetrazol‐5‐ylmethyl) benzo (TTB), 1D Zn‐based MOF (Zn‐TTB) effectively passivated defects, regulated crystal orientation, and enhanced device stability via self‐assembly (see Figure 7C).

Additive engineering in PSCs involves incorporating various additives to enhance film crystallization, minimize defects, and regulate interfaces. These additives facilitate homogeneous nucleation and crystal growth, which are crucial for achieving good surface coverage and desired crystal size. In turn, this process reduces electrical shunting, recombination, and trap states. ILs have emerged as promising alternatives to toxic solvents like DMF and DMSO. For instance, MAAc has been shown to improve crystallinity, reduce defect density, and enhance pore filling in perovskite films, leading to high‐performance solar cells [71]. Additionally, BAAc and MAFa have been effective in modulating crystallization and improving stability [74, 75]. Ammonium salt derivatives and polymers, with their unique functional groups, also play crucial roles in enhancing crystalline properties and surface morphology, leading to improved efficiency and stability of PSCs. Small organic molecules, such as fullerene derivatives and pyridine, are effective in passivating defects and enhancing device stability through efficient penetration into the perovskite lattice [87, 88]. MOFs have shown promise in stabilizing organic small molecules and improving device performance through defect passivation and crystal orientation regulation.

### 3.3 Solvent Engineering

**FIGURE 8:**
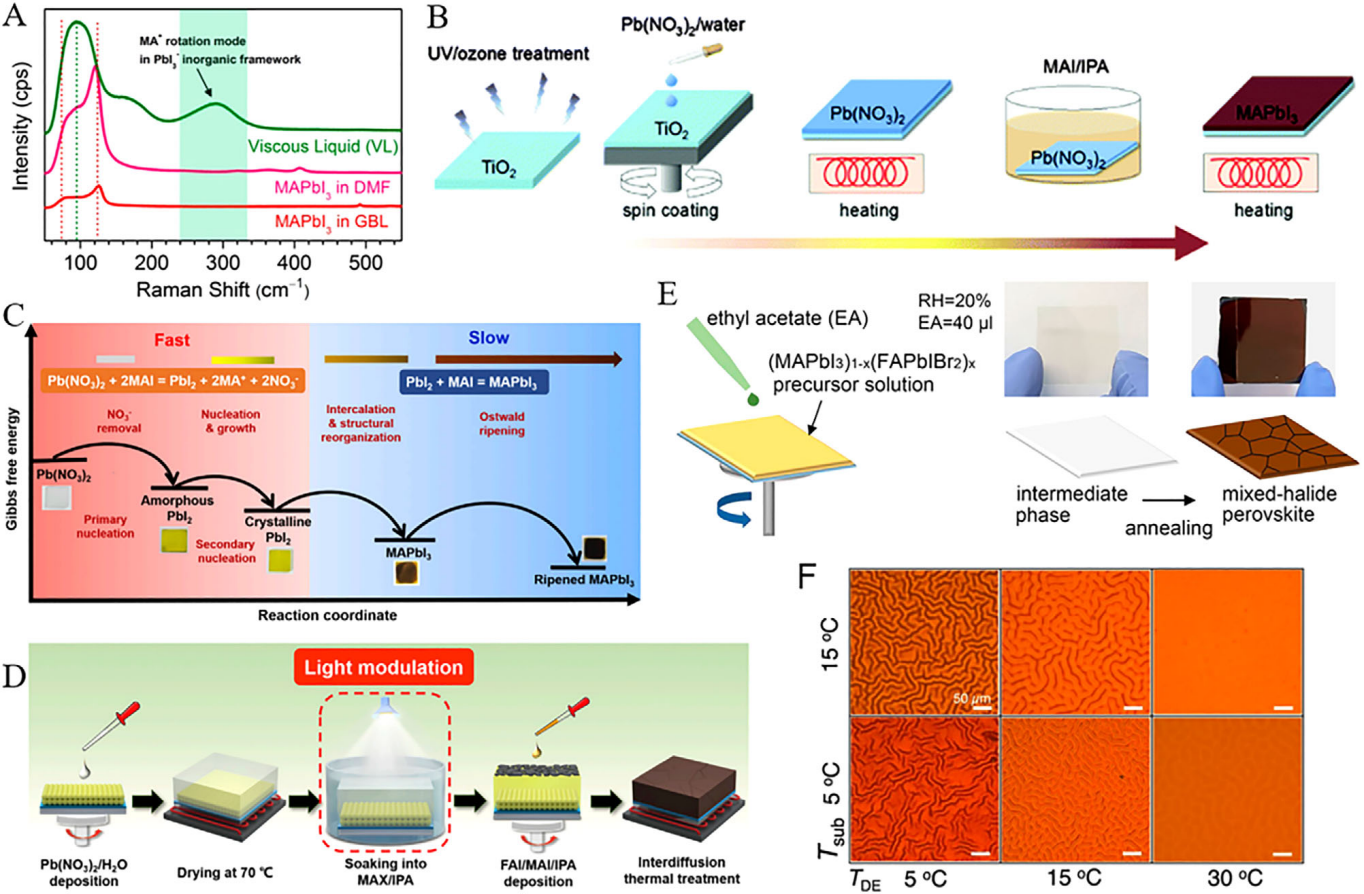
(A) Raman spectra of a viscous liquid were compared with those of MAPbI_3_ solution dissolved in GBL and DMF. Reproduced with permission [91]. Copyright 2019, American Chemical Society. (B) Method for fabricating a MAPbI_3_ layer using the Pb(NO_3_)_2_/H_2_O system. Reproduced with permission [94]. Copyright 2015, The Society. (C) Diagram illustrating the conversion process within the Pb(NO_3_)_2_ film. Reproduced with permission [97]. Copyright 2022, Elsevier. (D) Flowchart depicting the fabrication process of PSCs using a light modulation strategy. Reproduced with permission [97]. Copyright 2022, Elsevier. (E) Schematic illustrating the green solvent engineering process for depositing perovskite thin films. Reproduced with permission [100]. Copyright 2019, Elsevier. (F) Optical microscope images of FA_0.875_Cs_0.125_Pb(Br_0.125_I_0.875_)_3_ perovskite films captured at varying temperatures of *T*_DE_ and *T*_Sub_. Reproduced with permission [101]. Copyright 2021, Springer Nature.

Utilization of toxic solvents in the fabrication of perovskite films is a significant concern within the field. For example, the solution processing method, recognized for yielding high‐efficiency PSCs often relies on various toxic solvents such as DMF, DMSO, *N*‐methyl‐2‐pyrrolidone (NMP), dimethylacetamide (DMAc), 1,3‐dimethyl3,4,5,6‐tetrahydropyrimidin‐2(1H)‐one (DMPU), and tetrahydrofuran (THF) etc. [89] In comparison to commonly employed solvents for scaling up (such as DMF, DMSO, and NMP) acetonitrile (ACN) was considered a green solvent due to its lower boiling point to other non‐volatile solvents comparatively. For instance, Noel et al., [90] developed an industrially scalable solvent system by dissolving methylamine in acetonitrile with a rapid evaporation by one‐step spin‐coating. This approach led to the swift crystallization of perovskite material at room temperature with PCEs of 19.0% and 16.8% for conventional and inverted structures, respectively. Moreover, Jeong et al., [91] successfully deposited large‐area MAPbI_3_ films (exceeding 100 cm^2^) on FTO substrates using perovskite colloids in both the viscous liquid and the diluted ACN solution, as shown in Figure 8A. The resulting D‐bar coated MAPbI_3_ exhibited a highly (*hk*0) oriented crystalline structure throughout the film, featuring a tetragonal/cubic superlattice structure with an average PCE ≈17.82% of 100 cm^2^ MAPbI_3_ film. Similarly, Baral et al., [92] proposed PEACl as an additive to enhance the crystallinity and reduce defects in MHP films. Moreover, MAPbI_3_ PSCs precursors are cast from volatile and environmentally friendly solvents (MA/ACN) under ambient air conditions, resulting in an efficiency of over 20%. Although the addition of certain volatile components may enhance perovskite film crystallization at lower temperatures, most substances are flammable, posing safety risks during thin film fabrication. Furthermore, perovskite films processed at lower temperatures frequently exhibit inferior crystallinity and higher defect density. For example, Fang et al., [93] proposed blade‐coating without subsequent thermal annealing for highly crystalline perovskite films at room temperature under ambient air conditions (RH > 50%). By this, perovskite precursor by solvent engineering with 2‐methoxyethanol (2ME) was tailored with coordinated ammonium halide species, and thus uniform colloids, homogeneous nucleation, and rapid crystallization of the perovskite films led to an impressive PCE of 19.16%.

Despite water being recognized as a preferred natural and eco‐friendly solvent for material synthesis, its practicality is hindered by the low solubility of solid lead halides and the susceptibility of perovskite to decomposition in aqueous environments. In this aspect, Hsieh et al., [94] developed an alternative novel aqueous precursor system (Pb(NO_3_)_2_/H_2_O) to conventional (PbI_2_/DMF) in MAPbI_3_‐based PSCs (see Figure 8B). With controlled morphology and surface coverage of the Pb(NO_3_)_2_ film during the coating process, a PCE of 12.58% was attained. Furthermore, Shinde et al., [95] demonstrated the transformation of MAPbI_3_ from a homogeneous Pb(NO_3_)_2_ layer, and hence the formation of PbI_2_‐free perovskite crystals with larger grain sizes was attained through an intermediate ion‐exchange process. Subsequently, Hsieh et al., [96] further explored the charge recombination and trap distribution in high‐efficiency MAPbI_3_‐based PSCs fabricated using an aqueous Pb(NO_3_)_2_ solution method with higher trap density. Moreover, to enhance the photovoltaic performance of Pb(NO_3_)_2_/water‐based devices, efforts yielded an improved efficiency of 18.3% and a high $V_{\mathrm{oc}}$ of 1.1 V so far. In a recent study, Zhai et al., [97] developed a light modulation strategy to synthesize perovskite from a water‐solvent‐based precursor system using Pb(NO_3_)_2_/H_2_O. In this regard, Figure 8C,D reveals the light exposure with significantly enhanced conversion kinetics and shaped the microstructure of lead halide. Specifically, light can trigger the photo‐decomposition of PbI_2_ and facilitate the oxidation of iodide to tri‐iodide, processes crucial for enhancing the quality of resulting perovskites. Thus, the water‐processed perovskite demonstrated a record PCE of 23.74% with exceptional environmental stability.

Similar to water, alcohols were utilized for the fabrication of perovskite films, however, the dissolution of PbI_2_ was found weak in organic salt precursors by the two‐step deposition method. For example, Wang et al., [98] developed a facile fabrication approach with ultra‐dry IPA_100_ that produced uniform and loosely packed PbI_2_ films, and hence highly crystallized MAPbI_3_ films under ambient conditions resulted conversion efficiency of 16%. Being a polar protic solvent, ethanol is commonly used in perovskite layers due to its low boiling point, though it could be utilized as a primary solvent for perovskite precursors to realize the dissolution of lead iodide through Lewis acid‐base chemistry. Thus, a stable FAPbI_3_ perovskite precursor solution was proposed by Seok's group, and thus the solvent system dimethylacetamide (DMA) and alkylammonium chloride (RNH_3_Cl) in ethanol resulted in a dense thin film without antisolvent dripping [99]. Additionally, the length of the alkyl group in RNH_3_Cl significantly affects the uniformity of perovskite films. This improved uniformity contributes to enhanced performance, allowing PSCs based on SnO2 electrodes to achieve a remarkable PCE exceeding 25%.

Given the health and environmental risks associated with commonly used antisolvents like toluene or chlorobenzene, researchers are actively investigating green alternatives as highly efficient PSC devices. These green antisolvents are expected to enhance homogeneous nucleation by accelerating crystallization rates while minimizing residual solvent content. Green antisolvents are categorized based on their chemical nature and compatibility with organic precursors and perovskite solutions, such as alcohol‐based, ester‐based, and ether‐based alternatives. Being weak polar solvents, alcohol‐based anti‐solvents have contributed to the low‐efficiency devices by degrading organic moieties (MA or FA) in perovskite structures [102]. Furthermore, Liu et al., [103] introduced an environmentally friendly approach utilizing cetyltrimethylammonium chloride (CTAC) and IPA as non‐toxic anti‐solvents to effectively mitigate surface and grain boundary defects in hybrid perovskites. The high electronegativity of the chloride group readily passivated anion vacancies, while the more stable cetyltrimethylammonium group demonstrated the passivation of cation defects. This approach significantly reduced the charge trap density and extended carrier recombination lifetime, consequently yielding a remarkable PCE of 23.4% in fabricated cells. Ester and ether‐based green anti‐solvents like ethyl acetate (EA) and diethyl ether (DE) were proposed to produce efficient PSCs. Notably, EA enables faster carrier transport rates and fewer defects compared to traditional chlorobenzene (CB) solvents [104]. In addition, relatively high boiling point (77°C) and low volatility ensured a rapid crystallization of perovskite in the intermediate phase PbI_2_‐MAI‐DMSO at room temperature. Similarly, Zhang et al., [105] introduced EA as a green antisolvent in perovskite crystallization process that yields uniform and dense perovskite films as characterized by large grain size, minimized grain boundaries, and low defect density. Additionally, utilizing a low‐temperature (100°C) processed SnO_2_ electron transport layer (ETL) reveals the favorable interface contact with perovskite layer, and hence enhanced photoelectron extraction and transport efficiency with a champion PCE of 17.83%. Likewise, Kim et al., [100] presented a controlled morphology of perovskites by adjusting the compositions and EA volume under ambient humidity as shown in Figure 8E. By this approach, incorporating FAPbIBr_2_ into MAPbI_3_ matrix yielded grains up to ≈1.5 µm that reduce the trap density and charge recombination. Thus, stability was improved due to reduced moisture diffusion of grain boundaries and led PCEs of ≈20.93% and 19.51% for active areas 0.12 and 0.7 cm^2^, respectively. Notably, the EA humidity resistance was maintained even at 50% RH and hence resulting in a remarkable PCE of 20.11%. As a non‐polar solvent, ether‐based green solvents such as DE have been widely utilized in PSCs devices. For instance, Zhang et al., [106] demonstrated incorporating 50% FA^+^ into MAPbI_3_, using a solvent‐mediated phase transformation process with DMSO and DE, yielding a highly crystalline, stable, and compact perovskite morphology. This approach resulted in long carrier lifetimes, low trap state densities, and record PCE ≈21.8% in alkaline‐metal‐free MA_0.5_FA_0.5_PbI_3_ perovskite‐based PSCs. Thus, Kim et al., [101] proposed micro‐wrinkled perovskite layers that may enhance the photocarrier transport performance by controlled geometry of microscopic wrinkles through exploiting temperature‐dependent miscibility with DE (see Figure 8F). Perovskite films with wrinkled morphology formed at *T*_DE_  =  5°C exhibited higher PCE and better stability than those formed at *T*_DE_  =  30°C. In this aspect, the optimization of interfacial and additive engineering has resulted in achieving a peak PCE of 23.02%.

Solvent selection in the fabrication of perovskite films is a pressing issue within the field due to the utilization of toxic solvents like DMF, DMSO, NMP, DMAc, DMPU, and THF. Researchers have actively sought greener alternatives to mitigate environmental and health risks associated with these solvents. ACN has emerged as a promising alternative, demonstrating potential for high‐efficiency PSCs [91]. Moreover, aqueous precursor systems have been developed to promote environmental sustainability in perovskite fabrication. Furthermore, efforts have been directed toward exploring alcohol, ester, and ether‐based green antisolvents to improve PSCs performance while minimizing health hazards linked to traditional solvents like toluene and chlorobenzene. By delving into these green solvent and antisolvent alternatives, researchers aim to not only enhance the efficiency but also ensure the sustainability of PSCs devices.

### 3.4 Interface Engineering

**FIGURE 9:**
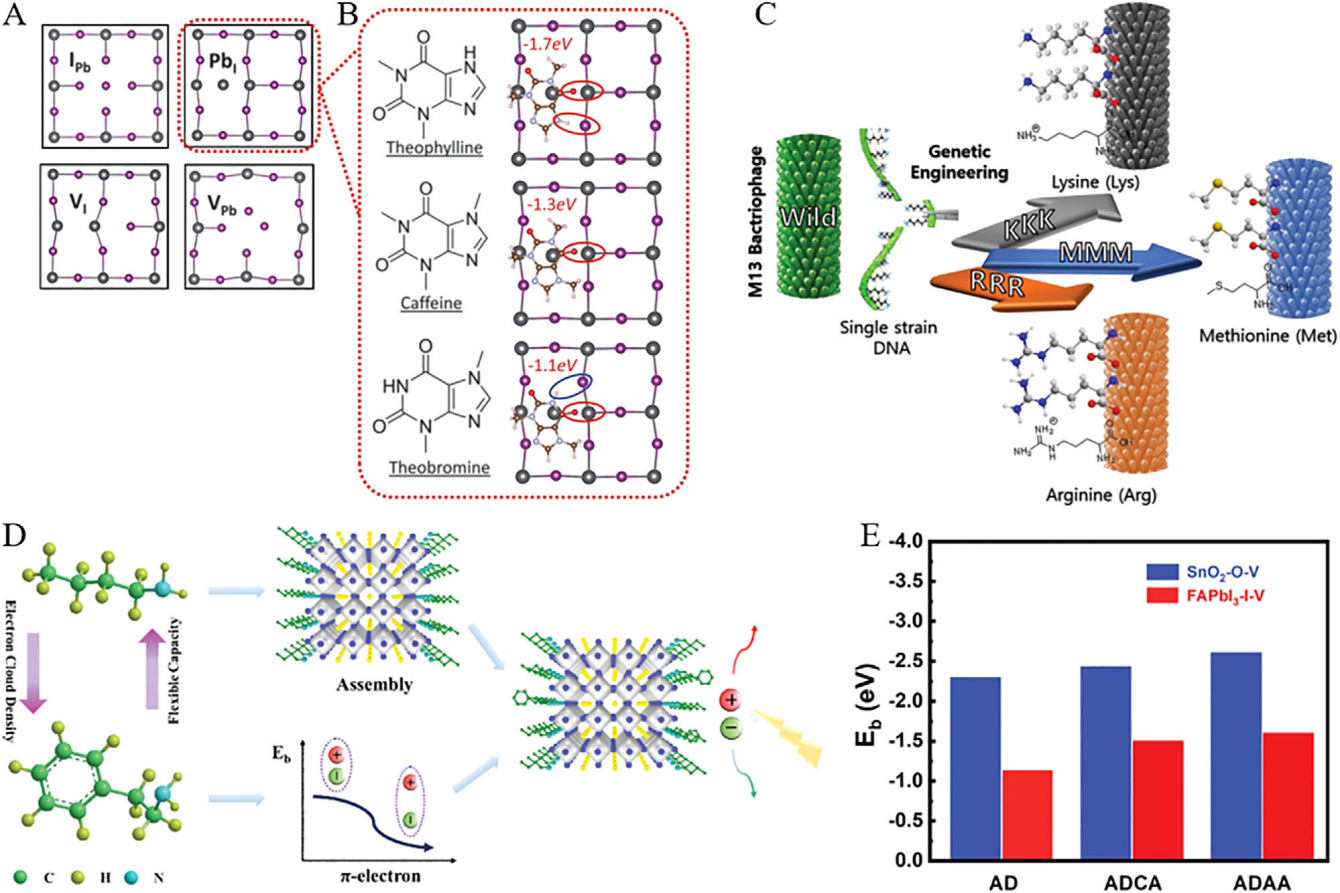
(A) Top‐down perspective displaying different categories of surface defects. Reproduced with permission [111]. Copyright 2019, The American Association for the Advancement of Science. (B) Schematic diagrams illustrating theoretical perovskite models with molecular surface passivation using theophylline, caffeine, and theobromine to target Pb_I_ antisite defects. Reproduced with permission [111]. Copyright 2019, The American Association for the Advancement of Science. (C) Diagram illustrating surface modifications resulting from viral genetic engineering, depicting the transformation from wild‐type to KKK‐, RRR‐, and MMM‐type viruses. Reproduced with permission [112]. Copyright 2019, The American Association for the Advancement of Science. Copyright 2021, John Wiley and Sons. (D) Schematic depiction of the mechanism involving the introduction of PEA^+^ into BA‐based layered perovskite. Reproduced with permission [114]. Copyright 2020, John Wiley and Sons. (E) Binding energies (*E*_b_) between oxygen vacancy defects in SnO_2_ and iodine vacancy defects in FAPbI_3_ when in contact with AD, ADCA, and ADAA molecules. Reproduced with permission [116]. Copyright 2022, John Wiley and Sons.

The interface between the perovskite layer and hole transport layer (HTL) is crucial for achieving high‐performance regular n–i–p PSCs. Solution‐processed polycrystalline perovskite films often exhibit increased defect concentrations in both bulk and surface regions. Specifically, defect density at perovskite films surface and interface is approximately 100 times higher than the bulk of perovskite films [107]. Recently, environmental friendly biomaterials have emerged as promising candidates for interface materials in both rigid and flexible PSC applications. These materials are interesting due to their low cost, diverse functional groups, and intriguing mechanical properties. Biomaterials such as bacteriophage, supramolecular biomaterials, zinc chlorophyll aggregates, DNA, and natural amino acids have been reported to significantly improve both PCE and the stability of PSCs [108]. In this scenario, Li et al., [109] employed cost‐effective and environmentally friendly zinc chlorophylls, Chl‐1 and Chl‐2, as hole‐transporting materials (HTMs) in one‐step solution‐processed CH_3_NH_3_PbI_3−_ *_x_* Cl*_x_*‐based PSCs. The Chl demonstrated ultra‐smooth surfaces, high hole mobility, appropriate energy levels, and efficient hole injection efficiencies, and hence PSCs based on Chl‐1 and Chl‐2 resulted PCEs of 11.44% and 8.06%, respectively. Furthermore, deoxyribonucleic acid (DNA) has emerged as a novel hole transport material that exhibits promising long‐range hole transport capabilities similar to a molecular wire in optoelectronics. For example, Hou et al., [110] demonstrated a core‐shell heterostructure of perovskite wrapped in cetyltrimethylammonium chloride‐modified DNA (DNA‐CTMA) via self‐assembly. This design enhances hole extraction and transport in bio‐photovoltaic devices and realizes an efficiency of ≈20.63%. Specially, perovskite grain boundaries were encased in the hydrophobic DNA‐CTMA shell to enhance device stability, retaining over 90% of initial efficiency after long‐term ambient exposure. Besides, molecular defect passivation strategies have influenced the ionic nature of lattice, involving interactions between functional groups and defects. In this aspect, Wang et al., [111] systematically investigated the role of activated functional groups, such as theophylline, caffeine, and theobromine for defect passivation. Optimal configurations facilitate the formation of hydrogen bonds between N─H and I due to the primary C═O binding with antisite Pb defects as shown in Figure 9A,B. Han et al., [112] utilized genetically engineered M13 bacteriophage as an eco‐friendly template for perovskite crystal growth as a passivation agent for PSCs. Through genetic manipulation, M13 bacteriophage enhanced the Lewis coordination with perovskite materials, particularly by amplifying specific amino acid groups, including lysine (Lys or K), arginine (Arg or R), and methionine (Met or M) (see Figure 9C). This effort indicated the genetically engineered M13 bacteriophage amplified with Lys and exhibited the PCE of 23.6% of PSCs.

In addition to the above, the presence of uncoordinated Pb^2+^ defects compromises the inherent stability of PSCs. Salt molecules like phenethylammonium iodide (PEAI), phenyltrimethylammonium bromide (PTABr), and *n*‐butylammonium iodide (BAI) with both anions and cations were employed to passivate the surface and interface defects in perovskite films [108]. In this regard, Yuan et al., [113] developed a fully automated spray‐coating technique combined with ultrathin‐film purification and thus colloidal CsPbI_3_ quantum dot (QD) thin films were fabricated. The enhanced carrier charge mobility and mitigated vacancy defects in QDs resulted in an improved photovoltaic performance and stability of the colloidal QDs (termed PTABr‐CsPbI_3_). Consequently, solar cells based on films spray‐coated with PTABr‐CsPbI_3_ QDs attained a PCE of 11% with 80% retention of initial PCE after 30 days. Zhou et al., [114] elucidated the synergistic effects between unsaturated alkylamine with π‐interactions and alkylamine in layered 2D perovskite materials. The alkylamine spacer cations were found to enhance precursor assembly, promoting the oriented growth of perovskite crystals. In this scenario, the incorporation of unsaturated alkylamine cations resulted in a reduced exciton binding energy that improved the carrier pathway in 2D perovskites (see Figure 9D). Combining both cations led to a significantly enhanced open circuit voltage in photovoltaic cells with an efficiency of 15.46%. Further, Jiang et al., [115] developed an organic halide salt PEAI for mixed perovskites FA_1−_ *_x_* MA*_x_* PbI_3,_ and hence the surface defects were effectively suppressed in perovskite polycrystalline films leading to an enhanced solar cell efficiency of 23.32%.

Coordination bonds typically exhibit greater stability compared to ionic bonds, however, Lewis bases can effectively form robust coordination bonds with uncoordinated Pb^2+^ defects [108]. For example, Liu et al., [107] introduced a novel strategy to passivate the interfacial defects as well as suppress stress in MA‐free perovskite films using a multi‐active‐site Lewis base ligand, namely (5‐mercapto‐1,3,4‐thiadiazol‐2‐ylthio)acetic acid (MTDAA). Experimental and theoretical analyses have realized strong chemical interactions between multiple active sites in MTDAA and undercoordinated Pb^2+^ at the surface or grain boundaries of perovskite films. Theoretical calculations revealed that multi‐active‐site adsorption is thermodynamically more favorable than single‐active‐site adsorption, irrespective of PbI_2_ or FAI terminations. MTDAA modification led to significantly reduced defect density, prolonged carrier lifetime, and effectively released interfacial residual stress. These efforts resulted in a remarkable enhancement of PCE from 20.26% to 21.92%. Notably, the C═O group has widely demonstrated the effective passivation of undercoordinated Pb^2+^ and/or halide vacancy defects through a strong coordination bond. Thus, Zhou et al., [116] introduced a steric hindrance‐based strategy for passivating buried interface defects and releasing stress at the SnO_2_/perovskite interface through adamantane molecules with C═O such as 2‐adamantanone (AD), 1‐adamantane carboxylic acid (ADCA), and 1‐adamantaneacetic acid (ADAA). These molecules effectively passivate interfacial defects and alleviate strain. As shown in Figure 9E, the strength of chemical interaction between C═O in molecules and perovskites or SnO_2_ decreases gradually with the distance from C═O to the bulky adamantane ring (AD > ADCA > ADAA). Experimental and theoretical analyses confirmed the steric‐hindrance‐dependent defect passivation effect, and thus interfacial chemical interaction strength, defect passivation, stress release, and device performance are inversely related to steric hindrance. Consequently, devices with incorporated ADAA have demonstrated promising PCEs of 22.83% and 23.18% via one‐step and two‐step approaches respectively. In addition, Liu et al., [108] reported a molecular locking strategy utilizing polydentate ulose‐3,5‐dibenzoate (DDPUD) green biomaterial to stabilize the top interface of perovskite films through effective defect passivation. This strategy elaborated a stable chemical approach on the perovskite film surface that effectively healed uncoordinated Pb^2+^ ions, halide vacancies, and/or I−Pb antisite defects. The implementation of the polydentate ligand facilitated diminished interfacial defects, prolonged carrier lifetimes, and released interfacial stress with enhanced moisture resistance. As a result, the incorporation of DDPUD yielded an impressive PCE of 24.47%.

In conclusion, optimizing the interface between the perovskite layer and HTL is critical for achieving high‐performance regular n–i–p PSCs. Polycrystalline perovskite films often suffer from increased defect concentrations, especially at the surface and interface, which can hinder overall performance. Biomaterials like bacteriophage, DNA, and natural amino acids offer promising avenues for enhancing both efficiency and stability [108]. Various strategies, including utilizing zinc chlorophylls as HTMs, incorporating DNA‐CTMA shells for perovskite passivation, and employing molecular defect passivation with functional groups, have shown significant improvements in PSC efficiency [110]. Furthermore, the use of salt molecules and organic halide salts has proven effective in suppressing surface and interface defects. Coordination bonds formed by Lewis bases with uncoordinated Pb^2+^ defects contribute to stability, while steric hindrance‐based approaches and molecular locking with biomaterials hold potential for defect passivation and stress release, ultimately resulting in remarkable enhancements in PCE.

## 4 Methods of Fabricating Large‐Area PSCs

To facilitate the development of PSCs from laboratory‐scale to industrial‐scale, advanced manufacturing techniques have been adopted for large‐area PSCs with impressive stability so far. In this section, we presented several wide methods (Table 2) for large‐scale fabrication of PSCs such as slot‐die coating, doctor‐blade coating, spray coating, and inkjet printing in detail. The following sections cover the prominent methods and insightful remarks on the practical and commercial‐scale production of PSCs.

**TABLE 2:** Advancements in large‐scale PSCs development under ambient air conditions.

Method	Device structure	Device area [cm^2^]/humidity	PCE [%]	Ref.
Slot‐die coating	ITO/PEDOT:PSS/perovskite/PCBM/PEI/Ag	0.3(60% RH)	11.4	[118]
	FTO/NiO*_x_*/perovskite/PCBM/TBAOH/Ag	0.09(50% RH)	14.3	[119]
	FTO/TiO_2_/MAPbI_3_/spiro‐MeOTAD/Au	0.125(40–50% RH)	14.5	[120]
	ITO/NiO*_x_*/MAPbI_3_/PCBM/BCP/Cu ITO/NiO*_x_*/CsFAPbI_3_/PCBM/BCP/Cu	0.14(20–40% RH) 0.14(20–40% RH)	16.06 17.33	[121]
	ITO/SnO_2_/perovskite/Spiro‐OMeTAD/Ag	0.07(40% RH)	14.55	[122]
	FTO/c‐TiO_2_/m‐TiO_2_/Cs_0.17_FA_0.83_Pb(I_0.83_Br_0.17_)_3_/spiro‐OMeTAD/Au	0.09	17.5	[123]
	ITO/SnO_2_/Perovskite/PTAA/Au	0.09(30–40% RH)	18	[124]
	FTO/TiO_2_/Perovskite/spiro‐OMeTAD/Au	0.025(25–60% RH) 1(25–60% RH)	18.1 14.4	[125]
	FTO/SnO_2_/perovskite/Spiro‐OMeTAD/Au	0.09(10% RH) 57.5(10% RH)	18.94 16.22	[126]
	FTO/NiO*_x_*/P3HT‐COOH/perovskite/PCBM/PEI/Ag	0.09(40–60% RH)	18.07	[12e]
	FTO/c‐TiO_2_/MAPbI_3_:F‐LYS‐S/Spiro‐OMeTAD/Au	2.25(40% RH)	21.08	[127]
	FTO/c‐TiO_2_/MAPbI_3_:F‐GLU‐S/Spiro‐OMeTAD/Au	2.25(40% RH)	21.44	[128]
Doctor‐blade	ITO/PTAA/SiO_2_ NPs/FA_0.83_Cs_0.17_Pb(I_0.87_Br_0.13_)_3_/PCBM/BCP/Ag	0.24(30–50% RH)	16.7	[132]
	ITO/SnO_2_/MAPbI_3_:MAAc/Spiro‐OMeTAD/Ag	0.064(45–82% RH)	20.34	[134]
	FTO/ TiO_2_/perovskite/Spiro‐OMeTAD/Au	47(in air)	14.7	[135]
	FTO/SnO_2_/(FAPbI_3_)_1−x_(MAPbBr_3_)_x_/spiro‐OMeTAD/Au	0.16(20% RH) 10(20% RH) 53.6(20% RH)	20.49 16.54 13.32	[138]
	ITO/SnO_2_/FA_1−_ *_x_* MA*_x_* Pb(I_1−_ *_y_* Br*_y_*)_3_/Spiro‐OMeTAD/Ag	0.064(40–50% RH) 1.03(40–50% RH) 10.93(40–50% RH)	23.14 21.2 17.54	[136]
Spray coating	ITO/SnO_2_/perovskite/Spiro‐OMeTAD/Au PET/SnO_2_/perovskite/Spiro‐OMeTAD/Au	0.13(10–30% RH) 0.29(10–30% RH)	18.5 16.15	[144]
	ITO/NiO*_x_*/MAPbI_3_/C_60_/BCP/Ag	6.25(30–40% RH)	17.18	[145]
	FTO/c‐TiO_2_/m‐TiO_2_/CsPbBr_3_/MoS_2_/C	1(in air)	4.12	[146]
	FTO/SnO_2_/Cs_0.19_FA_0.81_PbI_2.5_Br_0.5_/Spiro‐OMeTAD/Au	0.16(10% RH)	19.17	[147]
	FTO/SnO_2_/perovskite/Spiro‐OMeTAD/Au	0.15(40% RH)	19.42	[148]
	FTO/MeO‐2PACz/MAPbI_3_/C_60_/BCP/Ag	2.5(in N_2_)	20.8	[149]
Inkjet Printing	FTO/c‐TiO_2_/m‐TiO_2_/ZrO_2_/C/perovskite	0.16(in air)	9.53	[149]
	FTO/c‐TiO_2_/m‐TiO_2_/MAPbI_3_/C	0.059(in air)	12.07	[153]
	FTO/c‐TiO_2_/m‐TiO_2_/MAPbI_3_/C	0.32(30–50% RH) 52.4(30–50% RH)	13.07 10.07	[12a]
	PET/PEDOT:PSS/perovskite/PCBM/BCP/Ag	0.1(35% RH)	16.6	[151]
	PET/PEDOT:PSS/Cs_0.1_(FA_0.83_MA_0.17_)_0.9_Pb(I_0.83_Br_0.17_)_3_/C_60_/BCP/Ag	1(20–25% RH)	11.4	[156]
	FTO/TiO_2_/C_60_/Cs_0.05_MA_0.14_FA_0.81_PbI_2.55_Br_0.45_/Spiro‐OMeTAD/Au	0.04(in air) 1.01(in air)	19.6 17.9	[157]
	ITO/NiO*_x_*/perovskite/C_60_/BCP/Au	0.01(45% RH)	21	[158]

Abbreviations: F‐GLU‐S, artificial peptide‐sulfonyl‐γ‐AApeptide; F‐LYS‐S, artificial amino acid; MeO‐2PACz, ([2‐(3,6‐dimethoxy‐9H‐carbazol‐9‐yl)ethyl]phosphonic acid.; PET, polyethylene terephthalate; PTAA, Poly[bis(4‐phenyl) (2,4,6‐trimethylphenyl)amine;TBAOH, tetrabutylammonium hydroxide; TEACl, 2‐thiopheneethylammonium chloride.

### 4.1 Slot‐Die Coating

**FIGURE 10:**
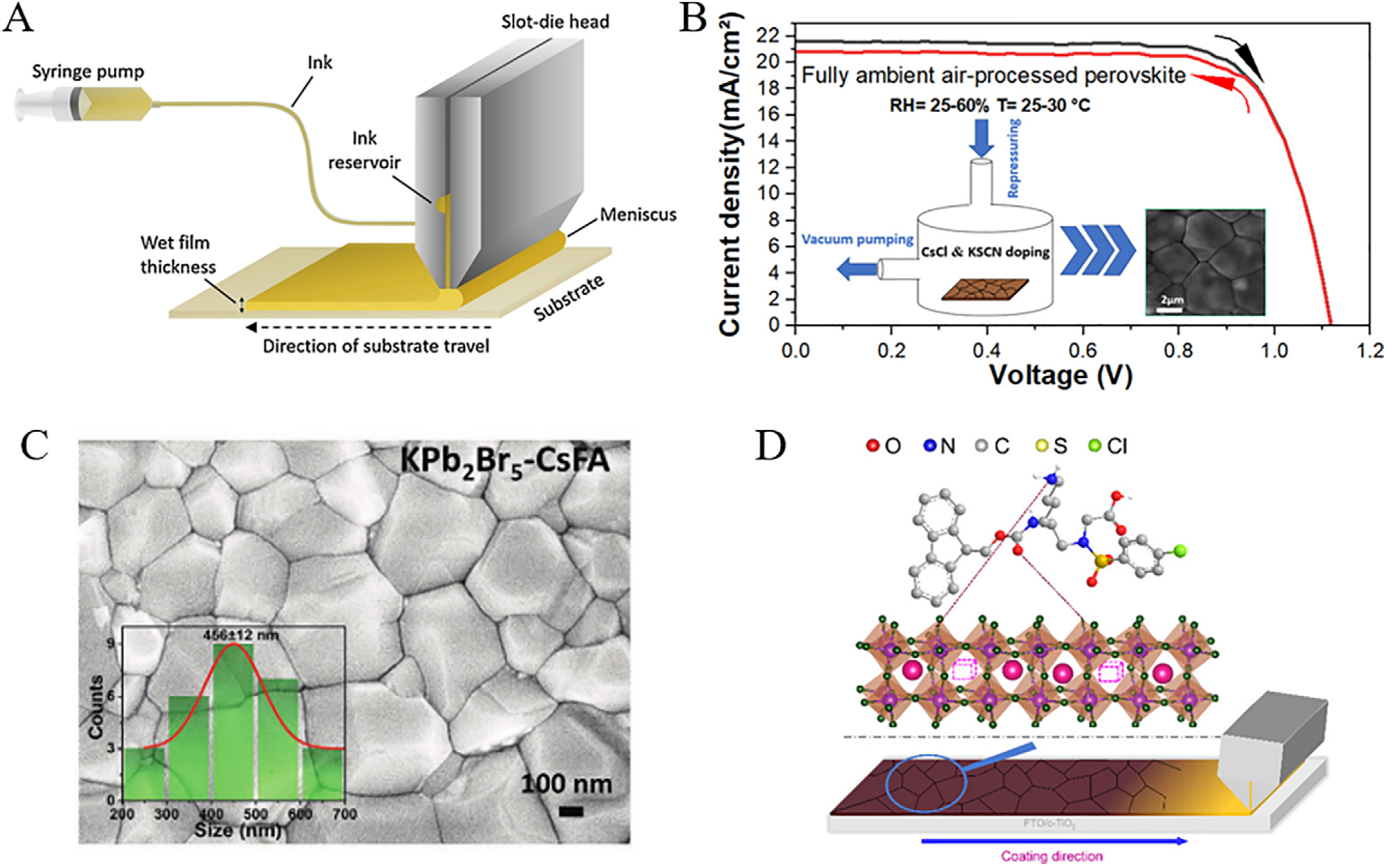
(A) Illustrated is the schematic depiction of a slot‐die coating procedure. Reproduced with permission [117]. Copyright 2020, Elsevier. (B) The addition of CsCl and KSCN resulted in an 18.1% PCE for the perovskite obtained through a fully air‐processed approach. Reproduced with permission [125]. Copyright 2023, American Chemical Society. (C) Top morphological FESEM portrayal exhibits the KPb_2_Br_5_‐CsFA perovskite film (with the inset showcasing a histogram of KPb_2_Br_5_‐CsFA perovskite grain size). Reproduced with permission [126]. Copyright 2022, John Wiley and Sons. (D) The interaction between F‐LYS‐S and MAPbI_3_ perovskite is depicted. Reproduced with permission [127]. Copyright 2023, American Chemical Society.

Slot‐die coating has emerged as a highly favorable technique for depositing all layers in the stack of PSCs due to its capability of minimal ink wastage. Typically, the thickness of deposited film can be precisely controlled by adjusting the ink flow rate through the coating head as shown in Figure 10A, and thus the thickness of film can be tuned ranging few nanometers to several tens of micrometers [117]. Recently, slot‐die coating techniques have emerged as versatile methods for depositing a variety of perovskite precursor solutions, thereby ensuring large‐area perovskite films with promising PCE. The integration of near‐infrared radiation methodology with slot‐die coating may lead to reduced annealing duration, however, the two‐step slot‐die coating technique revealed an efficiency of ≈11.4% with an active area of 0.3 cm^2^ under ambient conditions >60% RH [118]. Furthermore, Huang et al., [119] demonstrated the efficacy of substituting tetrabutylammonium hydroxide (TBAOH) for the conventional PEI in the functional modified layer of slot‐die‐coated PSCs, and hence a maximum PCE of 14.3% for PSCs with four layers of slot‐die coating with 0.09 cm^2^ effective area. In addition, the deposition on heated substrate at 90°C facilitated the formation of dense black perovskite films, avoiding the anti‐solvents or post‐treatment steps with a high PCE of 14.5% [120]. Except for surface roughness and high deposition temperatures, the numerous available defects on the coating surface restrict the performance of the coating. To address these challenges, Le et al., [121] reported a post‐treatment method known as vacuum‐assisted solution processing (VASP) to fabricate p–i–n type PSCs through slot‐die coating and thus impressive PCEs of 16.06% for MAPbI_3_ and 17.33% for CsFAPbI_3_ on a 0.14 cm^2^ area were realized. Similarly, PCEs of 11.7% and 14.9% were achieved for MAPbI_3_ and CsFAPbI_3_ micro‐modules (2.1 cm^2^), respectively. By employing a coordinated action of mixed solvents (DMF:NMP) and Cl‐based additives (MACl and FACl), Gao et al., [122] effectively controlled the crystallization process and attained a uniform morphology and high crystallinity of the perovskite films. Moreover, they successfully fabricated fully slot‐die‐coated PSCs in ambient air, achieving a maximum PCE of 14.55% with an effective active area of 0.07 cm^2^.

Besides, Bernard et al., [123] developed a one‐step slot‐die coating process combined with additives (MACl) and vacuum‐assisted solvent extraction to produce large‐area (5 × 10 cm^2^) Cs_0.17_FA_0.83_Pb(I_0.83_Br_0.17_)_3_ perovskite films with remarkable PCE of 17.5% on an active area of 0.09 cm^2^. In addition, Fievez et al., [124] employed a strategy of gas quenching and substrate heating coordination crystallization to facilitate rapid crystallization of slot‐die‐coated Cs_0.16_FA_0.84_Pb(I_0.88_Br_0.12_)_3_ perovskite on a 10 × 10 cm^2^ scale, achieving a PCE of 18% on an active area of 0.09 cm^2^ without the use of co‐solvents or additives. As shown in Figure 10B, potassium was found to effectively passivate defects and increase the grain size of perovskite, while SCN^−^ forms strong interactions with adjacent Pb atoms and enhances the chemical stability of perovskite with an optimal PCE of 18.1% (0.025 cm^2^) [125]. Rana et al., [126] achieved a uniform and highly crystalline Cs_0.15_FA_0.85_Pb(I_0.83_Br_0.17_)_3_ perovskite film by incorporating alkali metal salt CsPbBr_3_ and KPb_2_Br_5_ into the perovskite precursor ink (Figure 10C) and attained PCE of 18.94% on an active area of 0.09 cm^2^.

Additionally, a major issue contributing to the diminished efficiency of perovskite‐based devices is the substantial carrier transport loss occurring between the transport layer and perovskite layer. Glowienka et al., [12e] addressed this challenge by effectively mitigating the bulk defect density and carrier transport losses at the interface between the perovskite layer and HTL by the modified content of 2‐thiopheneethylammonium chloride (TEACl). Therefore, a PCE of 18.07% over an active area of 0.09 cm^2^ was achieved with excellent reproducibility. Furthermore, organic molecules with abundant functional groups such as carbonyl, carboxyl, hydroxyl, sulfonyl, etc., offer promising avenues for surface, grain boundaries, and electronic defect modification in perovskite materials. For example, devices based on slot‐die‐coated MAPbI_3_ with artificial amino acid (F‐LYS‐S) exhibited excellent performance (Figure 10D) and reached a PCE of 21.08%.[127] Abate et al., [128] further utilized multifunctional artificial peptide‐sulfonyl‐γ‐AApeptide (F‐GLU‐S) to modify the surface of perovskite thin films. The amino, carboxyl, and carbonyl functional groups of F‐GLU‐S interacted with MAPbI_3_ through Lewis acid–base interactions and significantly modified the iodine vacancies. As a result, the devices modified by F‐GLU‐S achieved a remarkable PCE of 21.44% with excellent stability characteristics.

### 4.2 Doctor‐Blade

**FIGURE 11:**
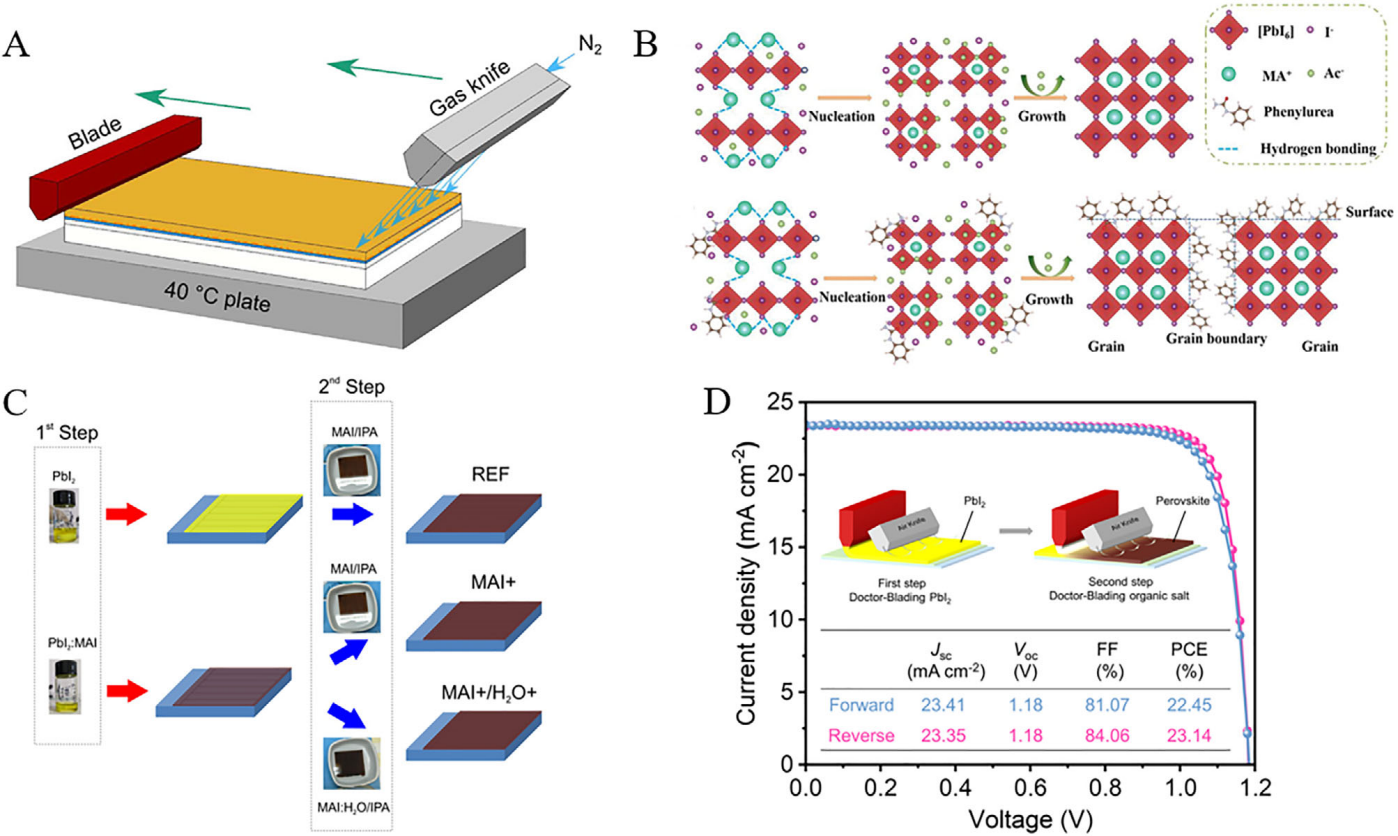
(A) Schematic representation demonstrating the process of blade coating facilitated by N_2_ gas stream. Reproduced with permission [132]. Copyright 2021, American Chemical Society. (B) Illustration of the mechanism of perovskite crystallization regulation facilitated by MAAc and MAAc‐phenylurea. Reproduced with permission [134]. Copyright 2023, John Wiley and Sons. (C) Schematic of the stepwise deposition process of CH_3_NH_3_PbI_3_ perovskite, detailing the modifications implemented to yield MAI^+^ and MAI^+^/H_2_O^+^ perovskites in the initial and subsequent stages, respectively. Reproduced with permission [135]. Copyright 2019, American Chemical Society. (D) *J*‐*V* characteristics of the structure of ITO/SnO_2_/FA_1−_ *_x_* MA*_x_* Pb(I_1−_ *_y_* Br*_y_*)_3_/Spiro‐OMeTAD/Ag prepared using a two‐step sequential doctor‐bladed method, achieved an efficiency of 23.14% under ambient conditions. Reproduced with permission [136]. Copyright 2023, Springer Nature.

The doctor‐blade coating technique has been demonstrated as a convenient, efficient, and low‐cost method for expanding perovskite thin films and PSC devices [129]. Currently, both one‐step and two‐step solution routes are employed to fabricate efficient PSCs [130]. The one‐step doctor‐blading technology, coupled with composition, solvent, and surfactant engineering, yields uniform and large‐area perovskite films with enhanced PCE and stability [131]. However, the frequent use of environmentally harmful precursor solvents such as DMF is still an arduous way to achieve scalable printing and large‐scale production of PSCs via the solution process. Küffner et al., [132] established the fabrication of inverted PSCs with dual cations without MA solely using DMSO in a one‐step blade‐coating process at low temperatures (Figure 11A). Remarkably, this approach yielded a PCE of up to 16.7% on an active area of 0.24 cm^2^. Nevertheless, the effectively controlled nucleation and growth processes during one‐step deposition of perovskite films in an ambient air environment make it challenging to control the morphology for large‐area films [133]. In this aspect, Li et al., [134] introduced a synergistic strategy by employing ionic liquid methylammonium acetate (MAAc) and benzylurea additive, and thus morphology and crystallization were modulated in MAPbI_3_ perovskite films (Figure 11B). The incorporation of MAAc facilitated uniform nucleation sites for perovskite formation, while the interaction between phenylurea and perovskite mediated by the C═O group and Pb^2+^ effectively regulated the crystal growth. Consequently, blade‐coated PSCs achieved an impressive PCE of 20.34% under environments exceeding 80% RH over an effective area of 0.064 cm^2^.

In a two‐step process, the PbI_2_ layer was initially deposited and subsequently converted into a perovskite film, followed by the deposition of organic ammonium salts. However, controlling the content of organic ammonium salts was challenging and led to an incomplete reaction between the PbI_2_ layer and organic ammonium salts. Matteocci et al., [135] tackled this issue by incorporating methylammonium iodide (MAI) into the PbI_2_ solution through a sequential deposition method (Figure 11C). This development led to improved morphology of the PbI_2_ film, consequently reducing crystal defects during the conversion process from PbI_2_ to perovskite. Notably, the substrate area of perovskite solar modules was increased from 5 × 5 cm^2^ to 10 × 10 cm^2^ and resulting in a PCE of 14.7% over an active area of 47 cm^2^. However, dense PbI_2_ films hinder the penetration and diffusion of organic salts, which inhibits the conversion of PbI_2_ to perovskite, whereas the porous PbI_2_ effectively reacts with organic salts [137]. Zhang et al., [138] achieved a continuous and uniform nanostructured PbI_2_ film via doctor‐blading and thus integrated TBP as a pore‐guiding additive in the PbI_2_ solution. Further, by introducing an optimal quantity of perovskite crystal seeds, they attained a maximum PCE of 20.49% over an area of 0.16 cm^2^. On the other hand, their perovskite solar modules demonstrated efficiencies of 16.54% for 5 × 5 cm^2^ modules and 13.32% for 10×10 cm^2^ modules. Wen et al., [139] manipulated the crystallization of PbI_2_ by introducing THTO (tetrahydrothiophene 1‐oxide), forming a PbI_2_‐THTO complex. The vertically packed PbI_2_ flaky crystals provided ideal nanochannels for the infiltration of ammonium salts, thus resulting in a remarkable PCE of 22.77% over an area of 0.16 cm^2^. Similarly, Chang et al., [136] utilized the modulating effect of MACl on perovskite growth to produce high‐quality, strongly oriented perovskite films via a two‐step sequential doctor‐blading process. As shown in Figure 11D demonstrate a champion PCE on PSCs ≈23.14% with an area of 0.064 cm^2^ for two‐step doctor‐bladed PSCs. However, PSCs with areas of 1.03 and 10.93 cm^2^ mini‐modules manufactured through this method have also achieved PCEs of 21.20% and 17.54%, respectively.

### 4.3 Spray Coating

**FIGURE 12:**
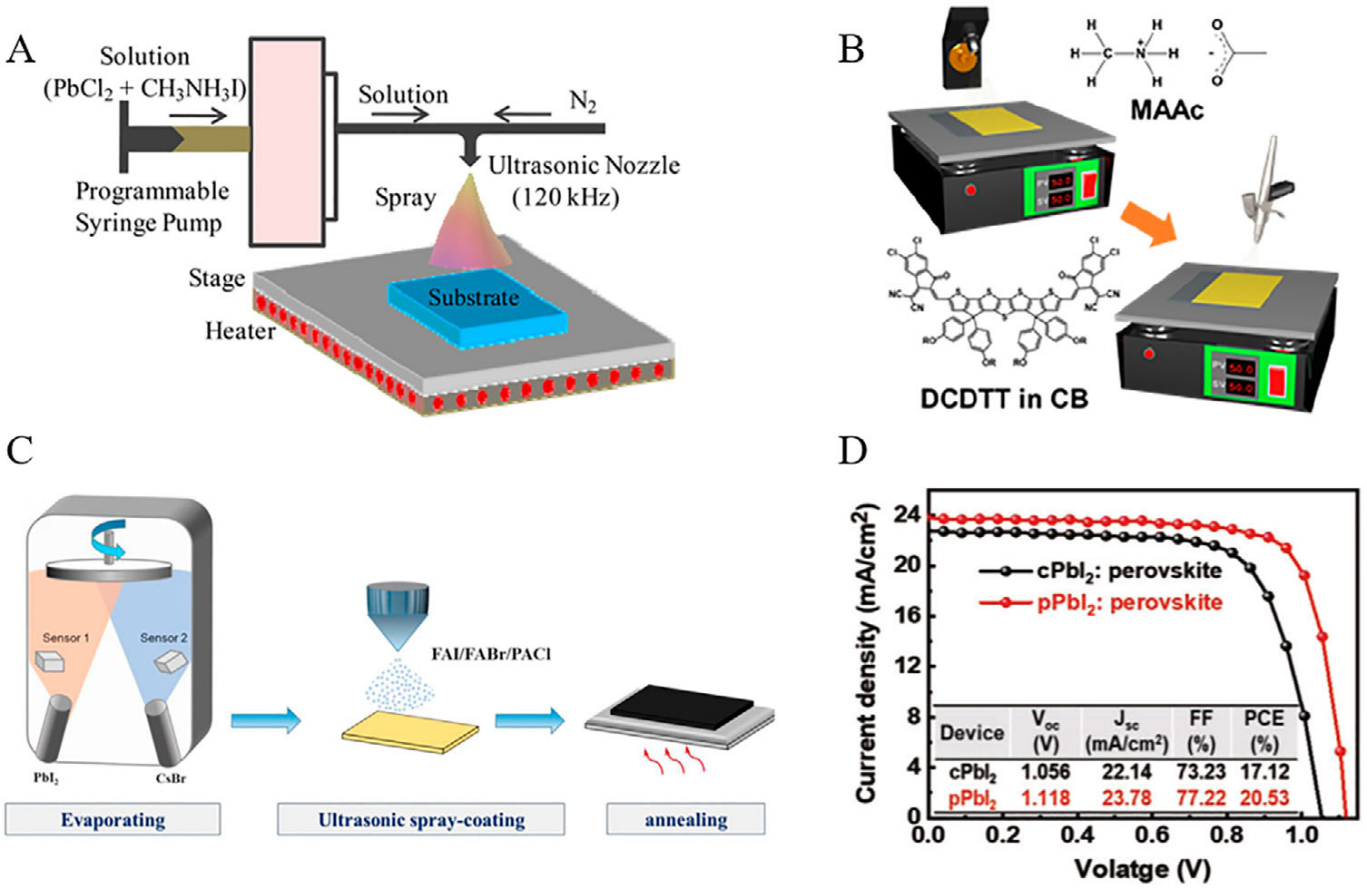
(A) Schematic illustration of the ultrasonic spray coating process. Reproduced with permission [140b]. Copyright 2015, American Chemical Society. (B) Illustration of the perovskite film deposition process accomplished via ultrasonic spray coating, with a precursor solution supplemented with an MAAc additive and DCDTT‐chlorobenzene antisolvent applied to the freshly formed perovskite film. Reproduced with permission [145]. Copyright 2022, American Chemical Society. (C) Schematic of the evaporative spray method for preparing perovskite films. Reproduced with permission [148]. Copyright 2023, Elsevier. (D) *J*‐*V* characteristic curves showing the performance of the fabricated devices. Reproduced with permission [12b]. Copyright 2023, John Wiley and Sons.

In recent years, spray‐coating technology has garnered widespread attention due to its low cost, suitability for large‐area fabrication, and compatibility with various substrates [140]. However, compared to spin‐coated films, spray‐coated films typically exhibit higher roughness and more pinholes, which severely compromise the performance of PSCs [141]. Barrows et al., [142] pioneered the fabrication of a planar‐structure solar cell using a one‐step ultrasonic spray coating technique and attained a PCE of 11.1% for an active area of 0.025 cm^2^. Similarly, Das et al., [140b] introduced the ultrasonic spray‐coating technique suitable to deposit perovskite on both rigid and flexible ITO substrates (Figure 12A), and hence PCEs of 13.0% and 8.1% were attained with effective areas of 6.5 mm^2^, respectively. Further, Tyagi et al., [143] employed a perovskite precursor solution by mixing lead‐containing precursors (PbAc_2_, PbI_2_, and PbCl_2_) with MAI to fabricate MAPbI_3_‐based PSCs via ultrasonic spray coating technique. The PCE increased to 15.7% for small devices and to 11.7% for 3.8 cm^2^ modules. Furthermore, Su et al., [144] successfully fabricated planar‐structured mixed cation‐based PSCs via spray‐coating technique and achieved a PCE of 18.5% (0.13 cm^2^) and 16.15% (0.29 cm^2^) on flexible substrates in comparison to the PCEs 15.07% and 13.21% for large‐area devices (1 cm^2^) on rigid and flexible substrates. Chen et al., [145] reported the synergistic effect of incorporating methylammonium acetate (MAAc) additive in the perovskite precursor solution and a non‐fullerene fused ring dicyclopentadithienothiophene‐based small molecule (DCDTT) as an additive in the antisolvent step (Figure 12B). Thus, a PCE of 17.18% for PSCs prepared at room temperature via ultrasonic spray coating was realized. These additives formed intermediate based on MAAc and DCDTT coordination and interacted with Pb^2+^, thereby improving the quality of MAPbI_3_ perovskite films and reducing grain boundaries and trap states.

Compared to the direct coating of perovskite precursors via a one‐step deposition regime, the sequential deposition technique offers enhanced control over film growth and improved uniformity and crystallinity. This method facilitates the fabrication of perovskite films on larger substrates and minimizes the formation of defects in films. Duan et al., [146] fabricated high‐quality CsPbBr_3_ thin films using spray‐assisted techniques and interface engineering in ambient air. They employed molybdenum disulfide quantum dots as an intermediate energy level at the CsPbBr_3_/carbon interface to effectively increase charge extraction and suppress electron‐hole recombination induced by perovskite grain boundary passivation, resulting in a PCE of 4.12% for CsPbBr_3_ solar cells with an active area of 1 cm^2^. Yu et al., [147] devised a reaction‐dependent tuning strategy by substituting IPA with *n*‐butyl alcohol (NBA) as the solvent in the spray‐coating process of FAI/Br solution. Specifically, the retarded‐drying‐time of NBA permitted sufficient spreading and penetration of the solution and enhanced the reaction between liquid droplets and solid film. This effectively inhibited the coffee ring effect and ensured the complete conversion of PbI2, ultimately resulting in an impressive PCE of 19.17% for Cs_0.19_FA_0.81_PbI_2.5_Br_0.5_. Furthermore, the average PCE of large‐area perovskite films (10 × 10 cm^2^) comprising 40 sub‐cells reached 18.33 ± 0.56%. Chen et al., [148] reported a two‐step evaporation spray coating method with an additive engineering strategy involving the incorporation of *n*‐propylammonium chloride (PACl) during spray processing (see Figure 12C). The propylammonium cation in PACl additives serves to passivate grain boundaries, while chloride ions can accelerate the transition of α‐phase perovskite, thereby enhancing the crystallinity of perovskite films. Consequently, efficient and stable methylammonium‐free PSCs were attained by this approach, which achieved the highest PCE of 19.42% with an active area of 0.15 cm^2^ modules. Tyagi et al., [12b] reported a method combining a spray‐assisted sequential deposition technique with propylene carbonate solvent additive for fabricating PSCs, achieving PCEs of 20.5% (Figure 12D) and 19.3% on effective areas of 0.09 and 1 cm^2^, respectively. Cassella et al., [149] achieved successful deposition of self‐assembled monolayer MeO‐2PACz for hole transport via ultrasonic spray coating and further explored optimization of spray‐coated perovskite films utilizing a gas knife quenching system to facilitate rapid solvent evaporation. The obtained optimum PCE reached 20.8% over an active area of 2.5 mm^2^.

### 4.4 Inkjet Printing

**FIGURE 13:**
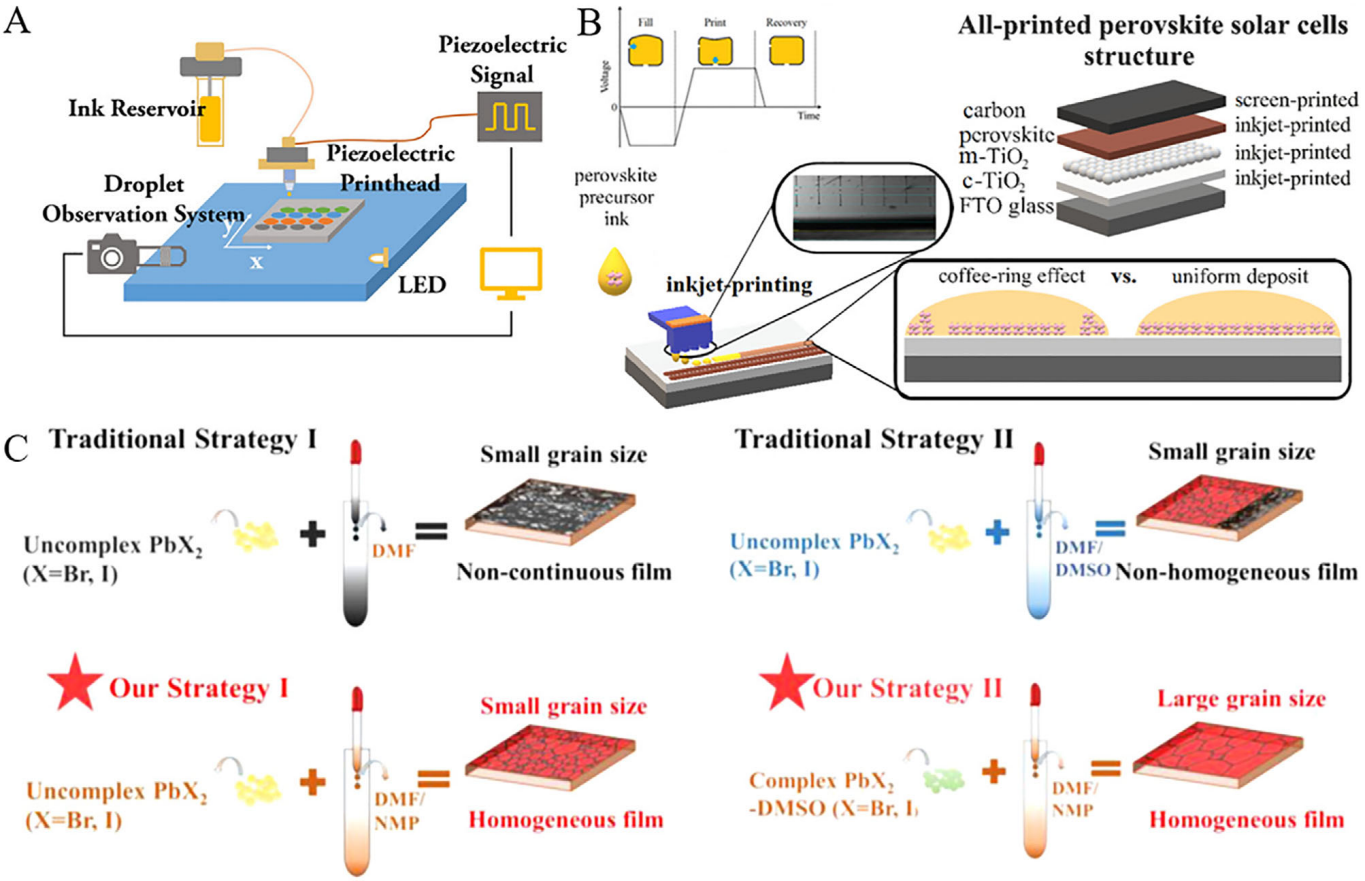
(A) Schematic illustration of the piezoelectric DOD inkjet printer alongside the droplet observation system. Reproduced with permission [151]. Copyright 2021, John Wiley and Sons. (B) Schematic depiction of inkjet‐printing processing for perovskite precursor inks and the structure of fully inkjet‐printed PSCs. Reproduced with permission [153]. Copyright 2022, John Wiley and Sons. (C) Schematic illustration of inkjet‐printed perovskite films showcasing four distinct strategies in ink formulation design. Reproduced with permission [157]. Copyright 2020, American Chemical Society.

Referring to the deposition of solution‐based materials, inkjet printing stands out as a digital technique renowned for its scalability, rapid yet precise material deposition, minimal waste generation, and capability to produce intricate patterns of printed inks at high resolutions [150]. Piezoelectric drop‐on‐demand (DOD) inkjet printing (see Figure 13A) exhibits remarkable precision in ejecting droplets of controlled size and velocity onto designated locations, facilitating meticulous regulation of the printing process [151]. Despite the limited exploration of inkjet‐printing processes for PSCs, significant progress has been made so far. In this aspect, Hashmi et al., [152] have pioneered the development of air‐processed permeable carbon‐based PSCs, achieving PCE reaching 9.53% on an active area of 0.16 cm^2^. However, the challenging “coffee ring” or “coffee stain” effect resulting from the uneven distribution of residues post‐solvent evaporation remains pertinent [151]. To tackle these issues, Chalkias et al., [153] introduced a novel strategy for regulating the concentration of perovskite precursor ink (Figure 13B), significantly reducing coffee ring defects during ambient air inkjet printing. Therefore, fully printed carbon‐based hole transport materials (HTM)‐free perovskite sub‐modules achieved an average PCE of 12.07% over an active area of 0.059 cm^2^ with excellent photovoltaic performance and strong stability against prolonged exposure to ultraviolet and solar light irradiation. Chalkias et al., [12a] further reported the formulation of an ink comprising eco‐friendly solvents and low‐concentration (0.8 M) perovskite precursors, yielding high‐quality perovskite thin films of carbon‐based HTM‐free materials prepared in ambient air with minimal coffee‐ring defects and a PCE of 13%. To resolve the dewetting on the bottom surface of substrate, incorporation of chlorine or bromine compounds was often employed to enhance ink wettability, thereby improving ink impregnation into porous materials. Gheno et al., [154] successfully developed fully inkjet‐printed MAPbI_3−_ *_x_* Cl*_x_* solar cells and realized a PCE of 10.7% under ambient conditions and at a low temperature (<90°C). Zhang et al., [151] introduced a thermal‐assisted inkjet printing process capable of directly depositing dense and uniform perovskite films onto PEDOT:PSS substrates in ambient air, achieving an optimal PCE of 16.6%. Different solvent compositions or precursor reaction rates can affect the nucleation/growth rate of perovskite films [155]. For instance, Senol Öz et al., [156] formulated ink for inkjet printing perovskite thin films under ambient conditions, achieving uniform, pinhole‐free perovskite thin films and obtaining a PCE of 11.4% on a 1 cm^2^ active area by adjusting the coordination environment of Pb^2+^ using the additive thiosemicarbazide. Furthermore, Li et al., [157] devised a novel mixed‐cation perovskite ink system, effectively retarding the crystallization rate of perovskite, achieving PCE of 19.6% (0.04 cm^2^) and 17.9% (1.01 cm^2^). In this new ink system (Figure 13C), the printing solvent consists of NMP and DMF, and PbX_2_‐DMSO (X = Br, Cl) complexes are used as printing precursors instead of PbX_2_, resulting in high‐quality perovskite films. Eggers et al., [158] demonstrated the inkjet printing of high‐quality triple cation perovskite layers with a thickness >1 µm, achieving a PCE exceeding 21% on an active area of 10.5 mm^2^.

In conclusion, slot‐die coating, doctor‐blade coating, spray coating, and inkjet printing represent diverse approaches for large‐scale PSCs fabrication, each with distinct advantages and drawbacks. Slot‐die coating stands out for its minimal ink wastage and precise film thickness control, ideal for large‐area deposition. Nonetheless, challenges like surface roughness and high deposition temperatures can hinder performance. Doctor‐blade coating offers convenience, efficiency, and low cost, yielding uniform and large‐area films, but concerns linger regarding environmentally harmful solvents and morphological control for extensive films. Spray coating boasts low cost, scalability, and substrate compatibility, yet it often results in rougher films with more pinholes compared to spin‐coated films, impacting PSC performance. Inkjet printing, renowned for precision and minimal waste, promises scalability and precise material deposition but must address challenges such as the “coffee ring” effect and residue distribution for optimal performance. Despite ongoing advancements to enhance efficiency and stability in each method, further research is essential to overcome inherent limitations and expedite widespread commercialization.

## 5 Summary and Outlook

PSCs have emerged as a promising solar technology, garnering significant attention due to their high efficiency, cost‐effectiveness, and simplified fabrication process. However, the highest‐efficiency devices have typically been obtained in a glovebox with small active areas due to the instability and inhomogeneity of perovskite films under ambient air conditions. Achieving high‐efficiency and stable large‐area PSCs, particularly under ambient air, remains a formidable challenge, which directly restricts their commercialization process. This review highlights the influence of environmental factors (moisture and oxygen) on PSCs' stability and offers a comprehensive analysis of degradation mechanisms. To tackle these challenges, various strategies for enhancing PSCs stability are explored, including compositional engineering, additive optimization, solvent modulation, and interface refinement. Compositional engineering adjusts perovskite material chemistry to improve stability, while additives enhance film crystallization and grain boundary properties. Solvent manipulation impacts film morphology and crystallinity, and interface engineering optimizes device interfaces. Additionally, the review outlines several large‐scale PSC preparation methods, including slot‐die coating, doctor‐blade coating, spray coating, and inkjet printing, each offering distinct advantages, crucial for advancing PSCs technology. Continuous refinement of these techniques will propel the evolution of PSCs technology, widening its applied scope in the realm of renewable energy.

Despite significant progress for PSCs prepared in ambient conditions, several challenges remain to be addressed, such as lead leakage, eco‐friendly fabrication, and reduced pollution. Therefore, future research directions could focus on the following aspects: (1) Developing environmentally friendly methods for fabricating PSCs is a crucial research direction. For instance, the application of green solvents, low‐temperature fabrication processes, and other technologies can reduce the environmental impact of the fabrication process while meeting the requirements for air processing. (2) Finding more stable perovskite materials is another important future direction. This can be achieved through the synthesis of novel perovskite materials or exploring other photovoltaic materials with better stability to address the degradation issue of current perovskite materials in air. (3) Developing effective encapsulation techniques can enhance the stability of PSCs in air. By designing efficient encapsulation materials and processes to block the ingress of moisture and oxygen from the surroundings, the perovskite materials can be protected from the influence of environmental gases. (4) Designing and optimizing novel electron transport layers (ETLs) and hole transport layers (HTLs) to improve the charge transfer efficiency and stability of PSCs. (5) Further research on nanoscale material control and interface engineering to enhance the performance and stability of PSCs in air. By controlling the structure and interface properties of nanomaterials, precise control and optimization of the properties of perovskite materials can be achieved.

In summary, research on the ambient fabrication of PSCs provides a promising pathway to enhance device stability and enable large‐scale production. Future research directions for air processing of PSCs will focus on environmentally friendly fabrication methods, the development of new materials, encapsulation techniques, novel transport layer materials, and nanoscale control, aiming to address the degradation issues of PSCs in air and further advance their commercial applications.

## Conflicts of Interest

The authors declare no conflicts of interest.
